# Anti-alcoholism drug disulfiram inhibits PANoptosis by blocking mitochondrial permeabilization in macrophages

**DOI:** 10.3389/fimmu.2025.1726408

**Published:** 2025-12-19

**Authors:** Ya-ping Li, Xin-jian Niu, Ge Zhang, On-kei Chan, Nuo Sun, Bo Hu, Zi-jian Shi, Dong-yun Ouyang, Xian-hui He, Qing-bing Zha

**Affiliations:** 1 Department of Clinical Laboratory, the Sixth Affiliated Hospital of Jinan University (Dongguan Eastern Central Hospital), Dongguan, China; 2 Department of Immunology and Microbiology, College of Life Science and Technology, Jinan University, Guangzhou, China; 3 Department of Nephrology, the First Affiliated Hospital of Jinan University, Guangzhou, China; 4 Department of Fetal Medicine, the First Affiliated Hospital of Jinan University, Guangzhou, China; 5 Center of Reproductive Medicine, the First Affiliated Hospital of Jinan University, Guangzhou, China

**Keywords:** disulfiram, hemophagocytic lymphohistiocytosis, macrophages, mitochondrial permeabilization, PANoptosis

## Abstract

**Introduction:**

PANoptosis is a form of inflammatory cell death that exhibits simultaneous activation of pyroptosis, apoptosis and necroptosis signaling. Disulfiram is a clinically used anti-alcoholism drug and can inhibit NLRP3 inflammasome activation and pyroptosis. However, it is unknown whether and how disulfiram interferes with PANoptosis and related inflammatory diseases.

**Methods:**

PANoptosis was induced in murine macrophages and related protein levels were assayed by immunoblotting. The effects of disulfiram on PANoptosis were assessed both in macrophages *in vitro* and in a mouse model of hemophagocytic lymphohistiocytosis (HLH) *in vivo*.

**Results:**

Mitochondrial permeabilization preceded lytic cell death upon PANoptosis and binding of GSDMD-NT, GSDME-NT and p-MLKL to mitochondria was linked to mitochondrial dysfunction, which was depending on cardiolipin synthesis in mitochondria. Intriguingly, disulfiram not only prevented mitochondrial permeabilization but also suppressed PANoptotic signaling activation in macrophages. Mechanistically, disulfiram prevented the binding of GSDMD-NT, GSDME-NT and p-MLKL from mitochondria to attenuate its permeabilization, release of its components and generation of reactive oxygen species. Furthermore, the assembly of PANoptosome was effectively blocked by disulfiram. In a mouse model of HLH, intraperitoneal administration of disulfiram substantially decreased systemic inflammation and mitigated liver, lung and kidney injury, which were accompanied by reduced activation of PANoptosis signaling in these organs.

**Conclusion:**

A previously unappreciated action of disulfiram to inhibit PANoptosis both *in vitro* and *in vivo* was discovered, thus repurposing this anti-alcoholism drug for the treatment of PANoptosis-related inflammatory diseases.

## Graphical Abstract

**Figure:**
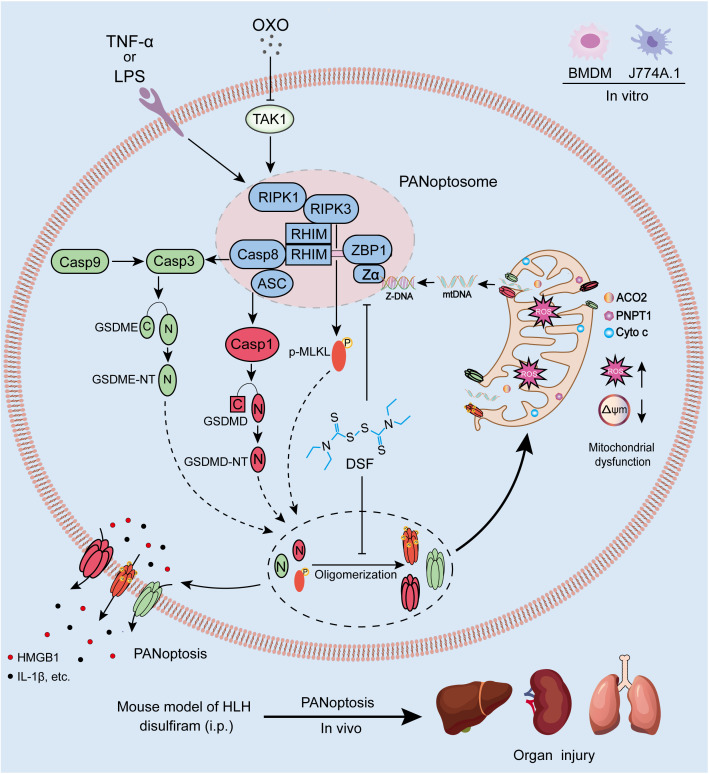

## Introduction

Inflammatory cell death (ICD) is cell death modalities that lead to lysis of cells and release of various damage-associated molecular patterns (DAMPs) including high mobility group protein B1 (HMGB1), ATP, and DNA. During ICD, the released DAMPs can further act on their respect receptors to trigger inflammation of other cells, thus playing critical roles in a broad spectrum of inflammatory diseases (1, 2). Several forms of ICD have been discovered over past decades, such as pyroptosis, necroptosis, and secondary necrosis of apoptosis (3, 4). PANoptosis a newly identified form of ICD that simultaneously manifests the hallmarks of pyroptosis (e.g., activation of caspase-1 and gasdermin D (GSDMD)), apoptosis (e.g., activation of caspase-3 and GSDME) and necroptosis (phosphorylation of mixed lineage kinase domain-like pseudokinase (MLKL)) (4, 5). The activated GSDMD (i.e., GSDMD-NT), GSDME (i.e., GSDME-NT) and phosphorylated mixed lineage kinase domain like pseudokinase (MLKL) (p-MLKL) can form oligomers and translocate into the plasma membrane to execute PANoptotic cell death, leading to the release of DAMPs and inflammatory responses (6, 7).

PANoptosis can be induced by different pharmacological stimulations or under different pathological conditions. For example, it has been revealed that the deficiency or inhibition of TGF-β activated kinase 1 (TAK1, also known as mitogen-activated protein kinase kinase kinase 7) induces the concurrent activation of the pyroptosis, apoptosis and necroptosis signaling in macrophages upon stimulation with lipopolysaccrides (LPS) or tumor necrosis factor α (TNF-α), indicating the induction of PANoptosis (8). Furthermore, PANoptosis can be elicited in macrophages by TNF-α in combination with interferon-γ (IFN-γ) (9). In addition, pharmacological agents or toxic natural products have also been found to induce PANoptosis (10, 11). Importantly, PANoptosis has been discovered to play important roles in many inflammatory diseases including severe coronavirus disease 2019 (COVID-19) and hemophagocytic lymphohistiocytosis (HLH) (1, 9, 12). PANoptosis has also been implicated in hemolytic diseases, in which heme plus pathogen-associated molecular patterns (PAMPs) drives this inflammatory process (13). Mechanistically, PANoptosis have been demonstrated to be mediated by a common molecular platform called PANoptosome that encompasses key components of pyroptosis, apoptosis and necroptosis signaling pathways (5, 12). Several forms of PANoptosome have been discovered under different stimulation circumstances (5). Upon induction of PANoptosis, the PANoptosome acts to mediate the activation of caspase-1, caspase-3 and RIPK3, which further trigger the production of GSDMD-NT, GSDME-NT and p-MLKL, respectively, to execute PANoptotic cell death (6, 7).

It is worth noting that mitochondrial dysfunction has been shown to be associated with lytic cell death during PANoptosis (14). Mitochondrial dysfunction can lead to retinal ganglion cell PANoptosis in glaucoma (15). Our recent studies showed that mitochondrial damage and thereby released components including cytochrome *c* and mitochondrial DNA (mtDNA) plays an important role in mediating lytic cell death during PANoptosis (16, 17). Furthermore, Z-DNA binding protein (ZBP1) plays an important role in promoting PANoptosis probably by recognizing Z-DNA that is derived from mtDNA (16). However, it is not known how mitochondria have been damaged. Of note, one recent study has shown an important role of GSDMD-NT in mediating mitochondrial permeabilization during pyroptosis (18). Additionally, GSDME-NT pores can permeabilize mitochondria to augment the activation of caspase-3 in cells expressing GSDME during apoptosis (19). The released components of mitochondria have been shown to play crucial parts in pyroptotic cell death during inflammasome activation by accelerating cell death (18). We speculated that permeabilization of mitochondria by PANoptotic executors (including GSDMD-NT, GSDME-NT and p-MLKL) may also play important roles during PANoptosis.

Disulfiram, a US Food and Drug Administration (FDA)-approved anti-alcoholism drug, has been shown to inhibit pyroptosis by targeting GSDMD-NT to block pore formation (20). A recent study further demonstrated that disulfiram also suppresses NLR family pyrin domain containing 3 (NLRP3) inflammasome activation by regulating the palmitoylation of this sensor (21). These studies highlight disulfiram as a potential drug to treat inflammatory disorders related to NLRP3 inflammasome and pyroptosis (20, 21). However, it is still not known whether and how disulfiram affects PANoptosis.

In this study, we demonstrated that mitochondrial permeabilization preceded lytic cell death during PANoptosis due to preferably translocation of GSDMD-NT, GSDME-NT and p-MLKL to mitochondrial membranes in a cardiolipin-dependent manner. The clinically approved drug, disulfiram, was able to inhibit PANoptosis by suppressing binding of these cell death executors to mitochondria and by attenuating PANoptosome assembly. Furthermore, disulfiram markedly mitigated systemic inflammation and multiple organ dysfunction in a mouse model of HLH, which was associated with decreased PANoptosis signaling in organs. Our data highlight a previously unappreciated action mechanism of old drug disulfiram in inhibiting PANoptosis and repurposing it for PANoptosis-related inflammatory diseases.

## Materials and methods

### Reagents and antibodies

Disulfiram (DSF, HY-B0240) and 5Z-7-oxozeaenol (OXO, HY-12686) were obtained from MedChemExpress (Monmouth Junction, NJ, USA). LPS (Escherichia coli O111:B4, L4391), Hoechst 33342 (B2261), propidium iodide (PI, P4170), DMSO (D8418), CF488-conjugated goat-anti-mouse IgG (SAB4600237), CF568-conjugated goat-anti-rabbit IgG (SAB4600084), Tween-20 (P1379), Tween-80 (P8074), and DL-dithiothreitol (DTT) (D0632) were purchased from Sigma-Aldrich (St. Louis, MO, USA). Murine TNF-α (315-01A) was obtained from PeproTech (Rocky Hill, NJ, USA). Poly(I:C) (HMW) (#tlrl-pic) were purchased from InvivoGen (San Diego, CA, USA). Dulbecco’s modified Eagle’s medium (DMEM) with high glucose (C11995500), fetal bovine serum (FBS, 10099141C), Opti-MEM (51985034), MitoSOX red mitochondrial superoxide indicator (M36008), SYTOX green (S7020), Lipofectamine RNAiMAX transfection reagent (13778075), Pierce classic IP kit (26146) and Pierce BCA protein assay kit (23227) were obtained from Thermo Fisher Scientific (Carlsbad, CA, USA). Phenylmethanesulfonyl fluoride (PMSF, ST505), TMRE (C2001) and ATP assay kit (S0026) were purchased from Beyotime (Shanghai, China). Specific antibodies against ASC (#67824), ASC AlexaFluor488 conjugated (#17507), COX IV (#11967S), ACO2 (6571), cleaved caspase-3 (#9664), cleaved caspase-8 (#8592), cleaved caspase-9 (#9509), HMGB1 (#3935), IL-1β (#12242), phospho(p)-MLKL (#37333), MLKL (#37705), PARP (#9532), RIPK3 (#95702), cytochrome c (#11940), β-tubulin (#86298), Na^+^K^+^-ATPase (#3010), and β-actin (#3700) were purchased from Cell Signaling Technology (Danvers, MA, USA). Antibodies against GSDMD (ab209845), GSDME (ab215191), pro-caspase1+p10+p12 (ab179515), and pro-caspase-8 (ab108333) were obtained from Abcam (Cambridge, UK). The antibody against ZBP1 (AG-20B-0010) was obtained from Adipogen AG (Liestal, Switzerland). The anti-PNPT1 antibody and anti-CRLS1 antibody (14845-1-AP) were obtained from Proteintech (Rosemont, IL, USA). Specific antibody against Z-DNA (Z22) (Ab00783-3.3) was purchased from Absolute Antibody (Oxford, UK). Anti-mouse F4/80-AlexaFluor647 (123122) and anti-mouse/human Mac2-AlexaFluor647 (125408) were purchased from BioLegend (San Diego, CA, USA). The antibody against 8-OHdG (sc-393871) was purchased from Santa Cruz Biotechnology (Santa Cruz, CA, USA).

### Animals

C57BL/6J mice (female, 6–8 weeks of age) were bought from the Laboratory Animal Center of Southern Medical University (Guangzhou, China). The mice used in this study were housed under stable conditions at 24 ± 2°C and a 12-h light/dark cycle and acclaimed for one week before experiments. The animal study was reviewed and approved by the Committee on the Ethics of Animal Experiments at Jinan University. Animal experiments were conducted in accordance with the National Research Council’s Guide for the Care and Use of Laboratory Animals.

### Cell culture

Mouse J774A.1 macrophage cell line was obtained from the Kunming Cell Bank of Type Culture Collection, Chinese Academy of Sciences (Kunming, China). The cells were maintained in complete DMEM medium (containing 10% FBS, 100 U/mL penicillin, and 100 µg/mL streptomycin) and cultured at 37°C in a humidified incubator with 5% CO_2_. The cells were sub-cultured every 2–3 days by using a cell scraper (541070, Greiner, Frickenhausen, Germany) to split cells. Before experiments, cells were cultured in complete DMEM medium overnight in 24-well plates at 9 × 10^4^ cells/well (0.5 mL) or in 6-well plates at 3.5 × 10^5^ cells/well (1.7 mL).

### Bone marrow-derived macrophages

Bone marrow-derived macrophages (BMDMs) were differentiated as previously reported (22). Briefly, C57BL/6J mice were sacrificed by cervical dislocation and sterilized with 75% ethanol. Bone marrow cells from hind femora and tibias of mice were cultured in BM-Mac medium (80% DMEM supplemented with 10% FBS, 100 U/mL penicillin, 100 µg/mL streptomycin, plus 20% macrophage-colony stimulating factor-contained medium from L929 cells) at 37°C in a humidified incubator of 5% CO_2_. After differentiation for 6 days in BM-Mac medium, BMDMs were incubated in six-well plates at 1.6 × 10^6^ cells/well (1.7 mL) or 24-well plates at 2.5 × 10^5^ cells/well (0.5 mL) with complete DMEM medium. The cells were ready for experiments after overnight incubation.

### Cell death assay

Cell death was measured as previously described (23). Briefly, cells in 24-well plates were treated with graded doses of disulfiram for 0.5 h, followed by indicated concentration of OXO for 1 h prior to TNF-α or LPS treatment for another 2 or 5 h, respectively. After indicated treatment, PI (2 µg/mL) and Hoechst 33342 (5 µg/mL) solutions were added into medium to stain dying cells and nuclei, respectively. The stained cells were immediately observed by live imaging using Zeiss Axio Observer D1 microscope equipped with a Zeiss LD Plan-Neofluar 20×/0.4 Korr M27 objective lens (Carl Zeiss, Göttingen, Germany). Fluorescence images were captured with a Zeiss Axiocam MR R3-cooled CCD camera controlled with ZEN software (Carl Zeiss). The percentage of lytic cell death was presented as the ratio of PI-positive cells (dying cells) to Hoechst 33342-positive cells (all cells).

### Precipitation of soluble proteins

Soluble proteins in the supernatant of cell culture were precipitated with 7.2% trichloroacetic acid plus 0.15% sodium deoxycholate, as previously described (16). The precipitated proteins were then washed three times with cold acetone and redissolved in a 2× sodium dodecyl sulphate-polyacrylamide gel electrophoresis (SDS-PAGE) loaded buffer, and the mature IL-1β, caspase-1p10 and HMGB1 were analyzed by Western blotting.

### Western blot analysis

For Western blotting, cultured cells and tissues were lysed in 2× SDS-PAGE sample loading buffer containing 200 µM dithiothreitol (DTT), and the cell lysates were ultrasonically treated to reduce viscosity. For detection of oligomerization of specific proteins, samples were prepared with 2× SDS-PAGE sample loading buffer without DTT. All samples were boiled for 5 min. The proteins were separated by SDS-PAGE and electrophoretically transferred to polyvinylidene fluoride (PVDF) membrane (#03010040001; Roche, Mannheim, Germany). The membranes were blocked with PBS containing 5% non-fat milk powder and 0.1% Tween 20 for 1 h. Subsequently, the membranes were incubated with an indicated primary antibody at 4°C overnight, followed by a secondary antibody coupled with HRP at room temperature for 1 h. Protein blots of interest were revealed by an enhanced chemiluminescence kit (BeyoECL plus kit; P0018S, Beyotime) and captured by X-ray film. ImageJ was used to analyze the density of each band.

### RNA interference

Small interfering RNA (siRNA) targeting the mouse Crls1 gene (encoding cardiolipin synthase 1, CRLS1) (5′-CCATGGACAATCCCAAATT-3′), *Zbp1* gene (encoding ZBP1) (5′-GCCTGCAACATGGAGCATA-3′) (16) and negative control siRNA (NC-siRNA) were designed and synthesized by RiboBio (Guangzhou, China). Knockdown of *Crls1* or *Zbp1* with 50 nM siRNA in BMDMs was performed by using Lipofectamine RNAiMAX transfection reagent according to the manufacturer’s protocol. Forty-eight hours after transfection, the cells were treated with OXO+TNF-α to induce PANoptosis, and then subjected to cell death assay, mitochondrial functional test or Western blot analysis, respectively.

### Immunofluorescence microscopy

Immunofluorescence staining was performed as previously described (16). In brief, cells were seeded in glass-bottom cell culture dishes overnight. After indicated treatments, the cells were fixed, permeabilized, blocked and then incubated with indicated primary antibodies at 4°C overnight. The cells were then incubated with CF647-conjugated goat anti-mouse IgG and CF568-conjugated goat anti-rabbit IgG at room temperature for 1 h, followed by staining with AlexaFluor488 conjugated anti-ASC at 4°C overnight, and then staining with Hoechst 33342 solution (5 μg/mL in PBS) to reveal the nuclei. Cell images were captured with a Zeiss Axio Observer D1 inverted fluorescence microscope and analyzed with the ZEN software (Carl Zeiss).

### Co-immunoprecipitation

The interactions among PANoptosome components were analyzed by Co-IP as previously reported (11). In brief, BMDMs in 6-well plates were pre-treated with disulfiram for 0.5 h, followed by treatment with OXO and TNF-α for 2 h in the presence or absence of disulfiram. After removing the culture medium, the cells were washed 2 times with ice-cold PBS and lysed with IP lysis/wash buffer (P0013; Beyotime) containing PMSF for 5–10 min on ice. Cell lysates were collected, transferred to pre-chilled tubes, and centrifuged at 13,000 rpm (4°C) for 10 min to obtain supernatants. Protein concentrations were determined using the Pierce BCA protein assay kit (#23227). Equal amounts of lysates (120 μg) were subjected to immunoprecipitation following the manufacturer’s protocol of the Pierce classic IP kit (#26146). After pre-clearing with control agarose resin, lysates were incubated overnight at 4°C with either mouse anti-pro-CASP8 antibody, RIPK3 or isotype control IgG (0.75 μg antibody per 120 μg lysates). Protein-antibody complexes were precipitated with Protein A/G-agarose and eluted by boiling for 10 min in 2× sample loading buffer containing DTT. Western blotting was used to analyze the abundance of CASP8, RIPK3, ASC, ZBP1 and β-actin in the eluted samples.

### Time course of cell death and mitochondrial membrane potential

MMP was detected with TMRE staining. Briefly, cells were seeded in 24-well plates overnight and then stained at 37°C for 15 min with TMRE (C2001, Beyotime) according to the instructions of the manufacturer. After washing, the cells were treated as indicated and were further stained with SYTOX green (100 nM) at 37°C for 15 min. Live-cell fluorescence images of TMRE and SYTOX green were acquired at Rhodamine and GFP channel, respectively, by using a Zeiss Axio Observer D1 inverted fluorescence microscope (Carl Zeiss). Mean fluorescence intensity was quantified with the ZEN software (Carl Zeiss).

### Time course of cell death and mitochondrial ROS

MitoSOX red was used to detect the mitochondrial superoxide levels (indicative of mtROS) in live cells according to the instructions of the supplier. In brief, cells were seeded in 24-well plates overnight and then treated as indicated. The cells were then stained with a combination of MitoSOX red (3 μM) and SYTOX green (100 nM) at 37°C for 15 min. Live-cell fluorescence images of MitoSOX red and SYTOX green were acquired at Rhodamine and GFP channel, respectively, by using a Zeiss Axio Observer D1 inverted fluorescence microscope (Carl Zeiss). Mean fluorescence intensity was quantified with the ZEN software (Carl Zeiss).

### Mitochondrial protein release assay

After indicated treatments, cells were collected and the cytoplasmic and mitochondrial fractions were separated according to the instructions of a cell mitochondria isolation kit (C3601, Beyotime). Western blotting was performed to detect cytochrome c, aconitase 2 (ACO2) and polyribonucleotide nucleoside transferase 1 (PNPT1) levels in cytoplasm and mitochondria, respectively.

### Plasma membrane extraction

As plasma membrane fraction extracted using the commercial kit (Membrane and cytosol protein extraction kit, P0033; Beyotime) contains mitochondrial membrane, we extracted plasma membrane from the cellular fraction after removing mitochondrial fraction using a cell mitochondria isolation kit (C3601, Beyotime). Western blotting was performed to detect indicated proteins in the isolated plasma membrane.

### Mitochondrial DNA release assay

To determine mtDNA levels in the cytoplasmic components of BMDM, cells were inoculated overnight in cell culture dishes (with 10 cm diameter) at a density of 1.0 × 10^7^/dish. After indicated treatments, separation of the cytosol and mitochondrial fractions was performed according to the instructions of the cell mitochondria isolation kit (C3601, Beyotime). The supernatant (the cytosolic fraction) was transferred to a new tube and the pellet was discarded. DNA was isolated from the cytosolic fraction using a Universal Genomic DNA Purification Mini Spin kit (D0063, Beyotime). Quantitative PCR (qPCR) was employed to measure mtDNA using TB Green Premix Ex Taq (Tli RNaseH Plus) (RR420A, Takara, Dalian, China) on a CFX96 Real-Time PCR Detection System (Bio-Rad, Hercules, CA, USA). mtDNA was quantified by qPCR using primers specific for COX-1 (encoding cytochrome c oxidase 1), and the mitochondrial D-loop region. Nuclear DNA encoding 18S rDNA and Tert, were used for normalization. Primer sequences are listed in **Table 1**.

**Table 1:** Sequences of primers for quantitative PCR.

Gene name	Primer sequence
*D-loop*	Forward: 5′-AATCTACCATCCTCCGTGAAACC-3′
	Reverse: 5′-TCAGTTTAGCTACCCCCAAGTTTAA-3′
*Tert*	Forward: 5′-CTAGCTCATGTGTCAAGACCCTCTT-3′
	Reverse: 5′-GCCAGCACGTTTCTCTCGTT-3
*COX-1*	Forward: 5′-GCCCCAGATATAGCATTCCC-3′
	Reverse: 5′-GTTCATCCTGTTCCTGCTCC-3′
*18S rDNA*	Forward: 5′-TAGAGGGACAAGTGGCGTTC-3
	Reverse: 5′-GTTCATCCTGTTCCTGCTCC-3′

### Cellular ATP assay

The cellular ATP levels were measured by using an ATP assay kit (S0026) according to the manufacturer’s instructions. Briefly, cells in 6-well plates were pretreated with disulfiram, followed by treatment with OXO and TNF-α for 2 h in the presence or absence of disulfiram. After treatments, the cells were lysed using the lysis buffer provided in the kit. The lysates were boiled for 3 min, followed by centrifugation at 12,000g for 5 min at 4°C. The supernatants were collected, mixed with ATP detection reagent, and transferred to a 96-well plate. The luminescence was immediately measured using a chemiluminescence-capable microplate reader (BioTek Synergy H1; Winooski, VT, USA). The ATP concentration was determined by comparing the readings with a standard curve.

### Poly(I:C)/LPS induced HLH model

A mouse model of HLH was established by sequentially administration of poly(I:C) and LPS as reported previously (9, 16). C57BL/6J mice (7–9 weeks of age) were randomly divided into 4 groups (total 20 mice: 5 mice for each group): Vehicle group, DSF group, poly(I:C)/LPS, and DSF plus poly(I:C)/LPS group. The mice in DSF group and DSF plus poly(I:C)/LPS group were orally administered with DSF (50 mg/kg body weight) formulated in vehicle sesame oil, which was based on our preliminary experiments and previous publications (20, 24). Three hours after DSF administration, the mice in poly(I:C)/LPS and DSF plus poly(I:C)/LPS groups were injected intraperitoneally (i.p.) with a single dose of poly(I:C) (10 mg/kg body weight). Six hours after poly(I:C) administration, the mice in poly(I:C)/LPS and DSF plus poly(I:C)/LPS groups were further injected (i.p.) with a single dose of LPS (1 mg/kg body weight). After 3 h, DSF group and DSF plus poly(I:C)/LPS group were orally administered with DSF (50 mg/kg body weight) again. Three hours after the second DSF administration, the animals were anesthetized with ethyl ether and blood samples were collected. The mice were then sacrificed by cervical dislocation and the liver, kidney and lung were collected. A part of the liver, kidney and lung tissues was used to prepare tissue lysates for Western blotting by using 2× SDS-PAGE loading buffer. The other part of the liver, kidney and lung tissues was fixed in a 4% neutral formaldehyde solution, embedded in paraffin, and subjected to conventional sectioning for hematoxylin and eosin (H&E) staining. The levels of serum creatinine, blood urea nitrogen (BUN), alanine aminotransferase (ALT), and aspartate aminotransferase (AST), were measured by using respective assay kits: serum BUN (E2020) and creatinine (E2038) were determined by spectrophotometry using respective assay kits (Applygen, Beijing, China) according to the manufacturer’s instructions; serum AST (BC1560) and ALT (BC1550) were assayed by using respective kits (Solarbio, Beijing, China) according to the instructions of the supplier. In a separate experiment, mice (total 30 mice: 10 mice per group) was administered with disulfiram (50 or 200 mg/kg body weight) together with poly(I:C)+LPS as above and observed every 6 h for 5 consecutive days to evaluate their survival.

### Histopathological scoring

To assess the severity of multi-organ damage in HLH model mice, a semi-quantitative analysis was performed on H&E-stained sections of the liver, kidney, and lung according to previous studies (25–27). All sections were evaluated in a blinded manner by investigators unaware of the experimental groups. The injury in each organ was scored on a scale of 0 - 3, as follows: 0 = none, 1 - 1.5 = mild (< 25% involvement), 2 - 2.5 = moderate (25% - 50% involvement), and 3 = severe (> 50% involvement).

### Detection of serum cytokines

Cytokines (IL-1β, IL-6, MCP-1, TNF-α, and IFN-γ) in sera of experimental mice were measured by cytometric bead array kits, a mouse IL-1β FlexSet kit (#560232) and a mouse inflammation kit (#552364), according to the instructions of the manufacturer (BD Biosciences, San Jose, CA, USA). Data were analyzed with a flow cytometer and related software (Attune NxT acoustic focusing cytometer, Thermo Fisher Scientific).

### Immunofluorescence staining of frozen sections

The liver, kidney and lung tissues were fixed with 4% paraformaldehyde for 24 h. Frozen sections with a thickness of 8 μm were routinely prepared, and the slides were stored at –80°C until staining. The tissue sections were sequentially equilibrated in PBS at room temperature for 10 min, soaked in sodium citrate buffer solution (10 mM, pH = 6.0) at 80°C for 30 min and blocked with PBS containing 5% normal goat serum and 0.1% Triton X-100 for 60 min. The sections were then incubated with the indicated antibodies at 4°C overnight, followed by incubation with the appropriate mixed fluorescent dye-labeled secondary antibodies for 1 h. The sections were covered by antifade mounting medium with DAPI and coverslips. Fluorescence images were captured by a Zeiss AxioCam MR R3 cooled CCD camera controlled with ZEN software (Carl Zeiss).

### Statistical analysis

All experiments were conducted three times independently. The data were presented as mean ± standard deviation (SD) and analyzed using GraphPad Prism 5.0 software (GraphPad Software, San Diego, CA, USA). Statistical significance for multiple comparisons was determined using one-way analysis of variance (ANOVA) followed by Bonferroni *post hoc* test. Mouse survival was analyzed using Kaplan-Meier survival curves and significance was evaluated by the log-rank (Mantel-Cox) test. A significance level of *P* < 0.05 was considered statistically significant.

## Results

### Permeabilization of mitochondria is an important stage preceding lytic cell death in macrophages during PANoptosis

**Figure 1:**
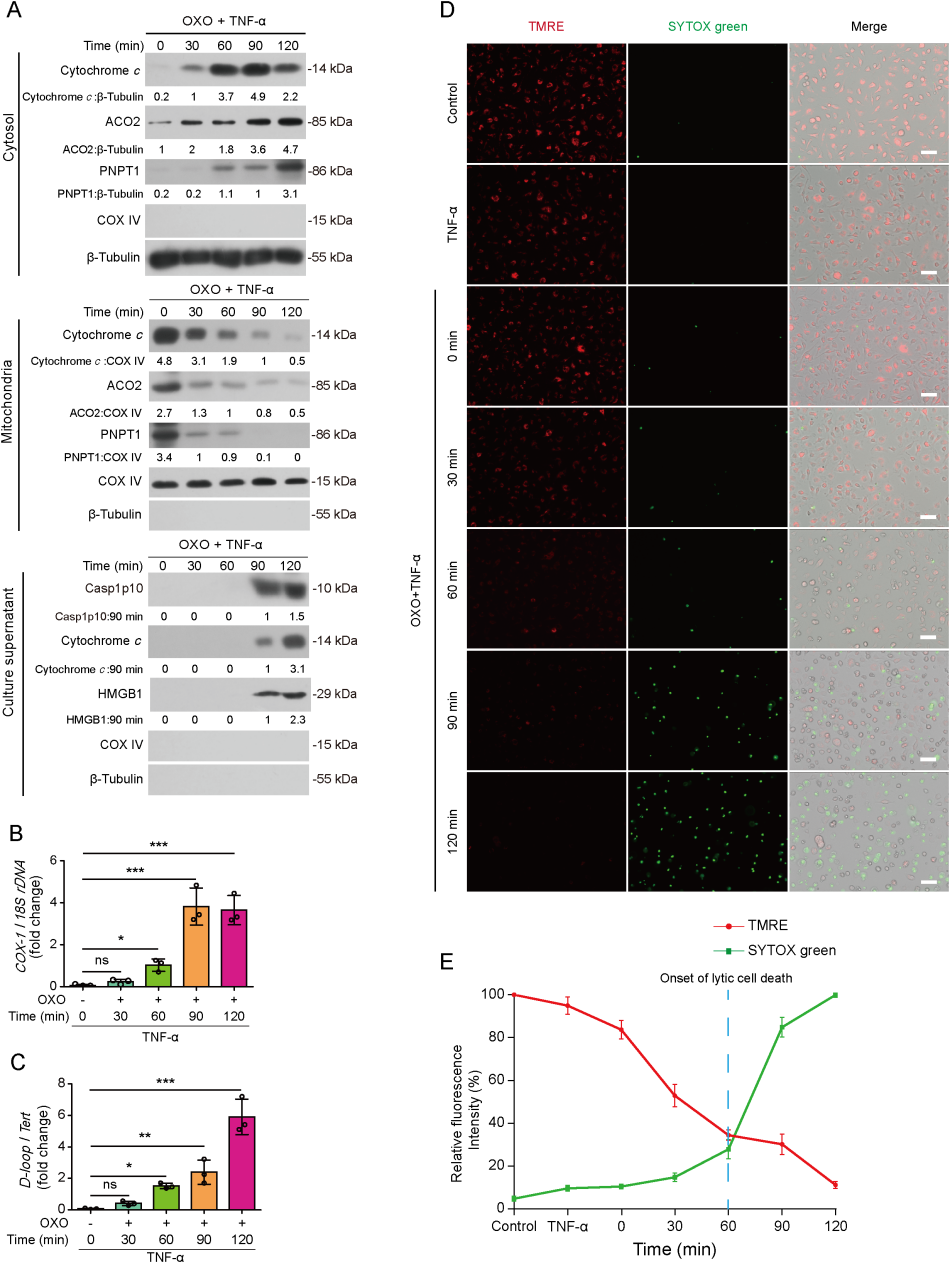
Mitochondrial permeabilization precedes lytic cell death during PANoptosis. Mouse bone marrow-derived macrophages (BMDMs) were treated with 5Z-7-oxozeaenol (OXO, 0.1 μM) for 1 h, followed by stimulation with TNF-α (5 ng/mL) for indicated time periods. **(A)** the levels of mitochondria-related proteins in mitochondria, cytosol and culture supernatants were detected by Western blotting. β-Tubulin was detected as internal control for the cytosol, which was not detectable in isolated mitochondria. COV IV was shown as internal control for mitochondria. The values under the blots represent their relative levels. **(B, C)** Mitochondrial DNA levels in cytosol were determined by quantitative PCR (qPCR). *P < 0.05; **P < 0.01; ***P < 0.001; ns, not significant. **(D, E)** Mitochondrial membrane potential probes (TMRE) and SYTOX green were used to measure temporal mitochondrial damage and lytic cell death, respectively. TMRE was stained for 0.5 h before OXO treatment, and SYTOX green was stained for 10 min after TNF-α treatment. Images were acquired by fluorescence microscopy. Scale bars, 50 μm **(D)**. The relative fluorescence intensity of TMRE and SYTOX green in 5 randomly chosen fields were quantified **(E)**. The vertical dotted line indicates the onset of lytic cell death as evidenced by Western blotting results in **(A)**.

Mitochondria have been shown to play a crucial role in PANoptosis (14–17), yet it is unknown how mitochondria have been damaged during PANoptotic cell death. In view of the role of GSDMD-NT in mediating mitochondrial dysfunction (18), we hypothesized that the PANoptotic executors, including GSDMD-NT, GSDME-NT and p-MLKL, might play a role in mitochondrial injury during PANoptosis. To this end, we adopted a PANoptosis cellular model in macrophages treated with TAK1 inhibitor, OXO, in combination with TNF-α with some data verified in cells treated with OXO plus LPS. PI incorporation assays showed that OXO+TNF-α for 2–3 h or OXO+LPS for 5–7 h induced 60-70% lytic cell death in primary BMDMs or J774A.1 macrophages. By using these cellular models, we assessed whether outer mitochondrial membrane (OMM) or inner mitochondrial membrane (IMM) had been permeabilized during PANoptosis induction. Western blot analysis showed that the mitochondrial proteins in the mitochondrial intermembrane space (IMS), including cytochrome *c* and PNPT1, were released into the cytosol as early as 30 min after OXO+TNF-α treatment whereas their levels in mitochondria were time-dependently decreased, indicating the permeabilization of OMM (**Figure 1A**). Similarly, matrix aconitase 2 (ACO2), but not IMM-associated COX IV, was increased in the cytosol as early as 30 min after the treatment whereas its mitochondrial level was time-dependently decreased, indicative of IMM permeabilization. Notably, cytochrome *c*, caspase-1p10 and HMGB1 were detected in cell culture supernatants at 90 min or longer time points, indicating plasma membrane disruption. Consistently, mtDNA was detected in the cytosol as early as 30 min after the treatment and its levels subsequently increased in a time-dependent manner (**Figures 1B, C**). Thus, these data suggest that mitochondrial permeabilization of OMM and IMM precedes the disruption of the plasma membrane during PANoptosis induction.

**Figure 2:**
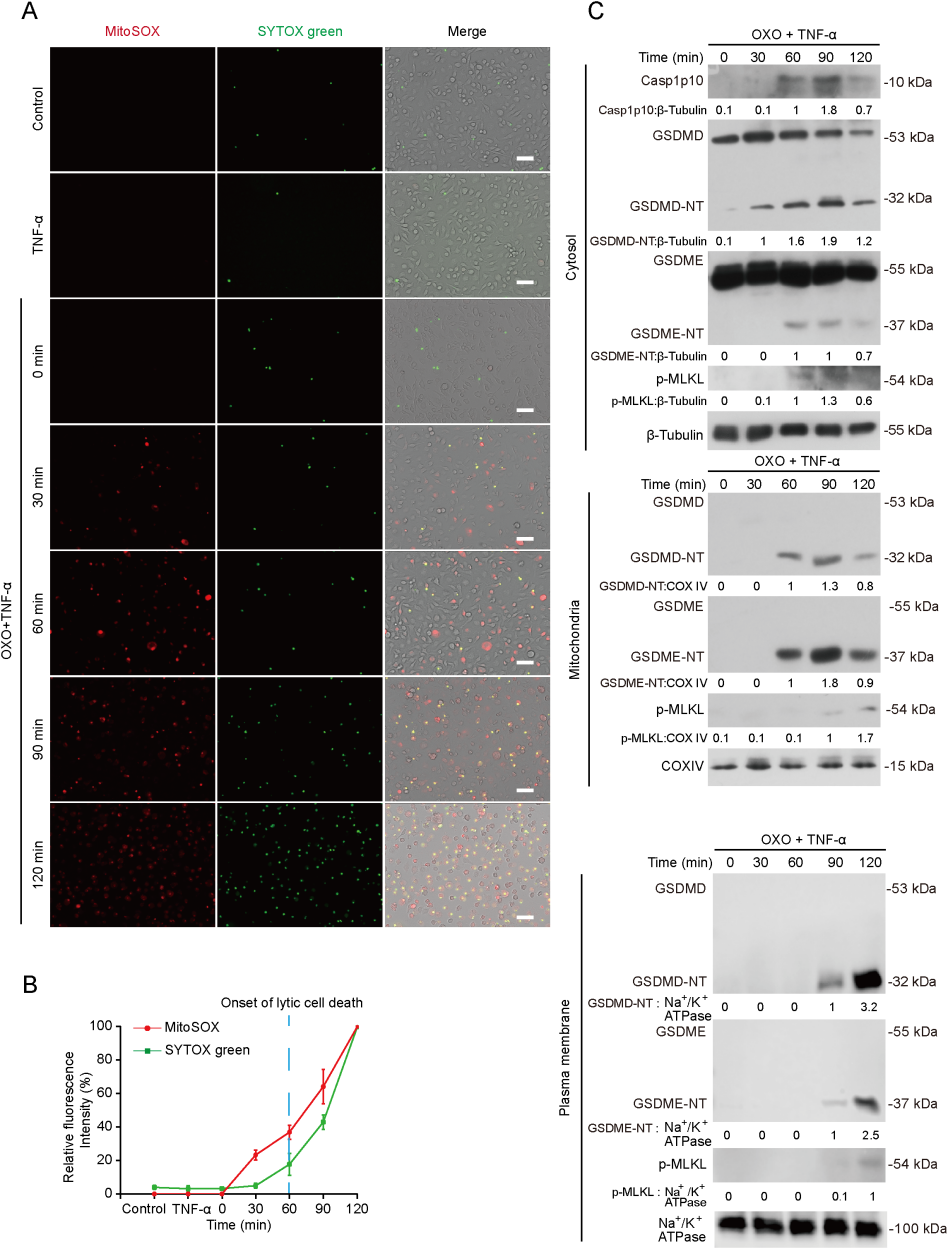
Mitochondrial reactive oxygen species (ROS) generation is earlier than lytic cell death and is associated with mitochondrial translocation of GSDMD-NT, GSDME-NT and p-MLKL during PANoptosis. BMDMs were treated with OXO for 1 h, followed by stimulation with TNF-α for indicated time periods. **(A, B)** Mitochondrial ROS (mtROS) were assayed by staining with MitoSOX and SYTOX green were used to reveal lytic cell death. SYTOX green and MitoSOX was stained for 10 min after TNF-α treatment. Images were acquired by fluorescence microscopy. Scale bars, 50 μm **(A)**. The relative fluorescence intensity of SYTOX green and MitoSOX in 5 randomly chosen fields were quantified **(B)**. The vertical dotted line indicates the onset of lytic cell death as evidenced by Western blotting results in **Figure 1a**. **(C)** Levels of PANoptosis hallmarks GSDMD-NT, GSDME-NT and p-MLKL in mitochondria, the cytosol and the plasma membrane were detected by Western blot analysis. COV IV and Na^+^/K^+^ ATPase were assayed as an internal control for mitochondria and the plasma membrane, respectively. The values under the blots represent their relative levels.

These results suggest a role of mitochondrial dysfunction in PANoptosis. In support of this notion, BMDMs were treated with OXO+TNF-α for various time periods to induce PANoptosis and stained with TRME (reflective of mitochondrial membrane potential) and SYTOX green (staining dying cells). The results showed that the decline of TMRE fluorescence was earlier than the increase of SYTOX green fluorescence (**Figures 1D, E**), indicating mitochondrial injury preceding lytic cell death. To further support this, MitoSOX (reflective of mtROS) and SYTOX green (indicative of dying cells) were used to stain the cells undergoing PANoptosis, and the results revealed that mtROS was generated earlier than lytic cell death (**Figures 2A, B**), which was also evidenced by the release of HMGB1, cytochrome *c* and casp1p10 (**Figure 1A**).

Together, these results indicate that mitochondrial dysfunction is prior to lytic cell death during PANoptosis induction, suggesting a potential role of mitochondria in this process.

### Mitochondrial damage is associated with cardiolipin-dependent translocation of GSDMD-NT, GSDME-NT and p-MLKL to mitochondria

We next explored whether GSDMD-NT, GSDME-NT and p-MLKL could bind to mitochondria during PANoptosis. Indeed, these PANoptosis executors were translocated to mitochondria as early as 60 min after OXO+TNF-α treatment (**Figure 2C**), whereas lytic cell death (**Figures 1D, E**), binding of these executors to the plasma membrane (**Figure 2C** bottom panel) and the disruption of the plasma membrane (**Figure 1A**, bottom) were only detected at 90 min or latter time point. These results indicated that PANoptosis executors preferably bound to mitochondria than to the plasma membrane and suggested that translocation of these executors might be associated with mitochondrial permeabilization and mtROS.

**Figure 3:**
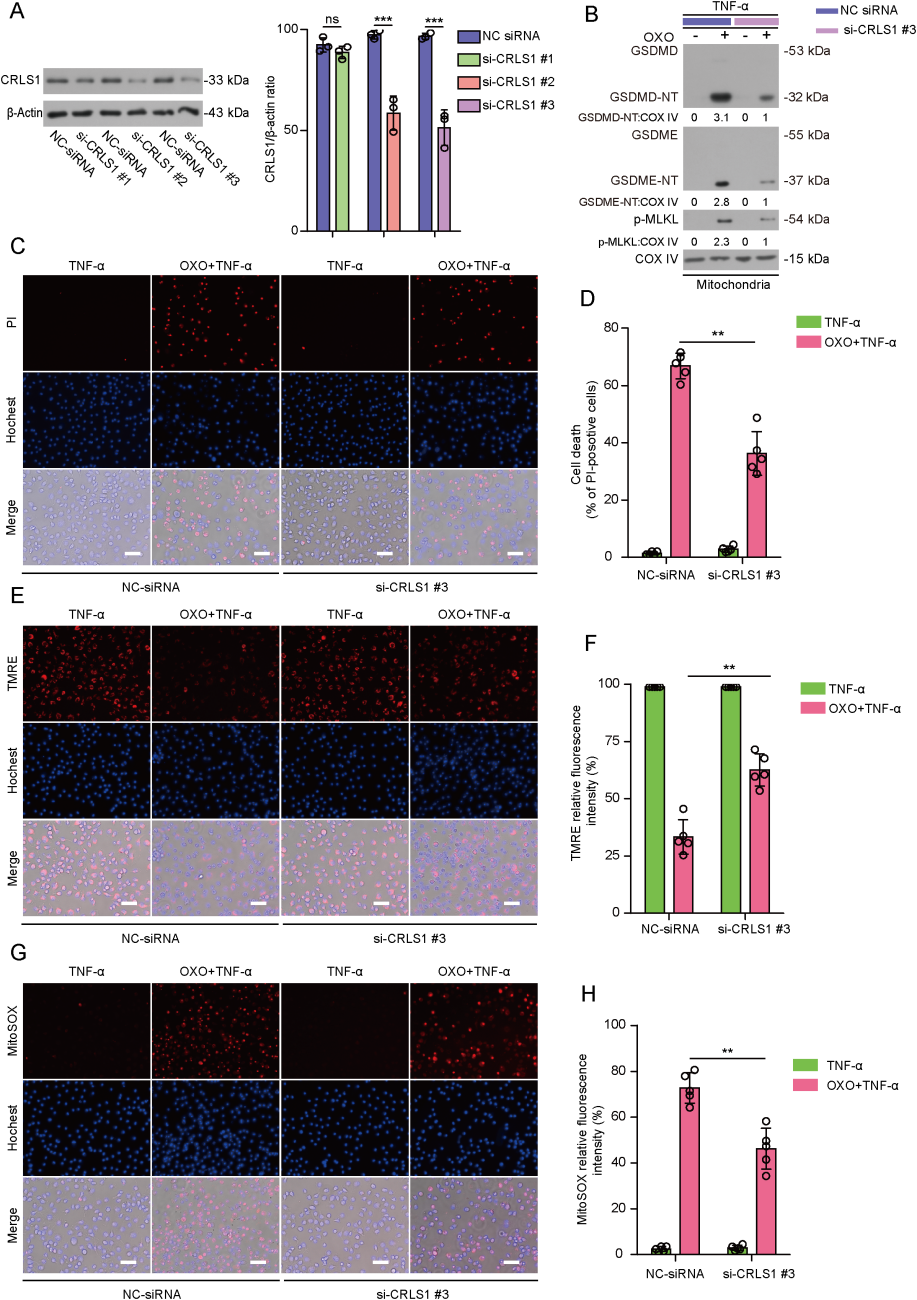
Knockdown of cardiolipin synthase 1 (CRLS1) attenuates PANoptotic cell death, mitochondrial binding of GSDMD-NT, GSDME-NT and p-MLKL, and mitochondrial dysfunction in macrophages. **(A)** The expression of CRLS1 in BMDMs was knocked down with three specific siRNAs (si-CRLS1 #1, #2 and #3) in comparison with the same NC-siRNA, and Western blot analysis was used to assay CRLS1 expression in BMDMs 48 h after siRNA transfection (**(A)**, left panel). The knockdown efficiency of si-CRLS1 was quantified and normalized to β-actin. Data are shown as mean ± SD (**(A)**, right panel). si-CRLS1 #3 exhibited the highest knockdown efficiency and was used for the following experiments. **(B)** Cells were treated with OXO for 1 h, followed by stimulation with TNF-α for 2 h, mitochondrial binding of cellular PANoptosis hallmarks GSDMD-NT, GSDME-NT and p-MLKL was detected by Western blotting. COV IV was detected as an internal control for mitochondria. The values under the blots represent their relative levels. **(C, D)** Analysis of lytic cell death in BMDMs by propidium iodide (PI) (red, staining dying cells) staining **(C)**. PI-positive cells in 5 randomly chosen fields were quantified and percentage of cell death is defined as the ratio of PI-positive over all cells (revealed by Hoechst 33342) **(D)**. **(E, F)** Mitochondrial membrane potential was analyzed by staining with TMRE **(E, F)**. **(G, H)** Mitochondrial ROS generation was assayed by staining with MitoSOX **(G, H)**. Images were acquired by fluorescence microscopy. Histograms showing quantitative analyses. Data are shown as mean ± SD (*n* = 5). Scale bars, 50 μm. **P < 0.01; ***P < 0.001; ns, not significant.

GSDMD-NT and GSDME-NT bind more strongly to cardiolipin than to other phospholipids (28–30), while p-MLKL can also bind cardiolipin (31). Given that mitochondrial cardiolipin has a crucial role in GSDMD-NT-mediated mitochondrial dysfunction and pyroptosis (18), we assessed whether cardiolipin synthesis was necessary for mitochondrial injury and lytic cell death during PANoptosis. To this end, we inhibited cardiolipin synthesis in mitochondria by knocking down cardiolipin synthase 1 (CRLS1), which is a crucial enzyme responsible for the synthesis of cardiolipin in mitochondria (32). Western blotting showed that the CRLS1 level was reduced ~50% by si-CRLS1 #3 (**Figure 3A**), which was used for the following experiments. After the knockdown of CRLS1, the levels of GSDMD-NT, GSDME-NT and p-MLKL in mitochondria were markedly reduced as compared to negative control (NC)-siRNA-treated cells during PANoptosis induction (**Figure 3B**). In line with this, PI staining showed that lytic cell death in CRLS1-knocking down macrophages was significantly decreased when compared with NC-siRNA-treated cells (**Figures 3C, D**). Furthermore, mitochondrial membrane potential in CRLS1-knocked down cells was increased when compared with control cells as revealed by TMRE staining (**Figures 3E, F**). Consistent with this, CRLS1 knockdown significantly reduced the production of mtROS as measured by MitoSOX staining (**Figures 3G, H**). Together, these results indicate that mitochondrial cardiolipin is required for translocation of GSDMD-NT, GSDME-NT and p-MLKL to mitochondria, thereby mediating mitochondrial dysfunction and lytic cell death during PANoptosis.

### Inhibition of PANoptosis by disulfiram is linked to suppression of mitochondrial permeabilization

**Figure 4:**
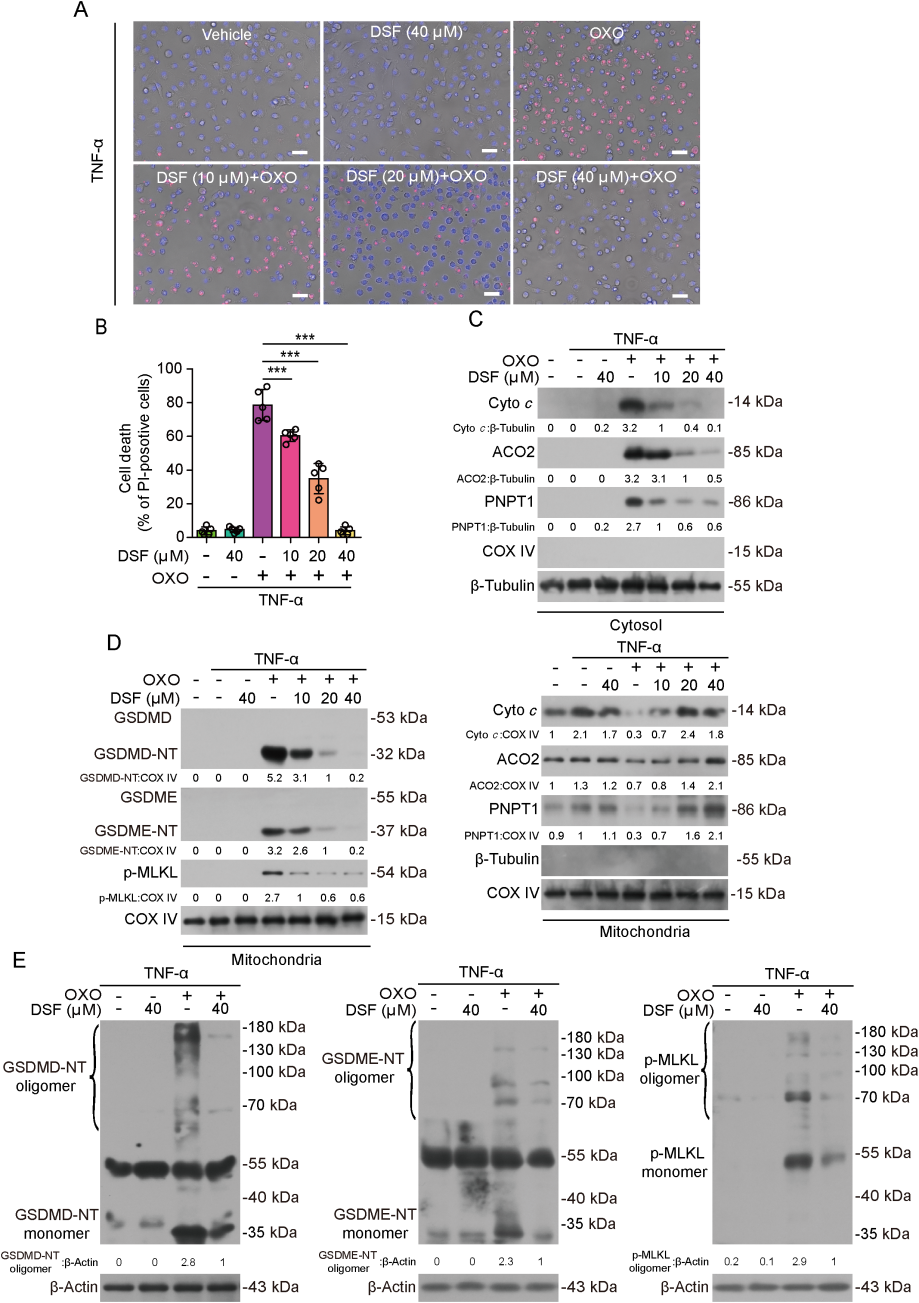
The inhibition of PANoptosis by disulfiram is associated with suppression of GSDMD-NT, GSDME-NT, and p-MLKL translocation to mitochondria and mitochondrial permeabilization in macrophages. BMDMs were pretreated with or without disulfiram (DSF) for 0.5 h, and then treated with OXO for 1 h, followed by stimulation with TNF-α for 2 h in the presence or absence of DSF. **(A, B)** Lytic cell death was measured by staining with PI (red, staining dying cells) and Hoechst 33342 (blue, staining all nuclei). Fluorescence and bright-field images were captured using fluorescence microscopy **(A)**. Histograms showing quantitative analysis of cell death **(B)**. Data are shown as mean ± SD (*n* = 5). Scale bars, 50 μm. ***P < 0.001. **(C, D)** The levels of mitochondrial protein in mitochondria and in the cytosol were detected by Western blotting, and the inhibitory effect of DSF on mitochondrial permeability caused by PANoptosis was analyzed **(C)**. The inhibition of PANoptosis by disulfiram is associated with suppression of GSDMD-NT, GSDME-NT, and p-MLKL translocation to mitochondria **(D)**. β-Tubulin and COV IV were detected as internal controls for the cytosol and mitochondria, respectively. **(E)** Western blotting of cell lysates from BMDMs was performed under a non-reducing condition to detect the oligomerization of GSDMD-NT, GSDME-NT, and p-MLKL, respectively. The values under the blots represent their relative levels.

Given that disulfiram can target GSDMD-NT to inhibit pore formation in the membrane (18) and that our above-mentioned data showing a potential role of GSDMD-NT, GSDME-NT and p-MLKL in mitochondria injury, we hypothesized that disulfiram might also target GDSME-NT and p-MLKL thereby preventing mitochondrial permeabilization and PANoptosis. Indeed, accompanying the inhibition of lytic cell death in BMDMs stimulated with OXO+TNF-α (**Figures 4A, B**) or OXO+LPS (**Supplementary Figures S1A, B**), disulfiram dose-dependently attenuated the release of IMS proteins cytochrome *c* and PNPT1 as well as matrix ACO2 (**Figure 4C**, **Supplementary Figure S1C**), indicating suppression of permeabilization of OMM and IMM, respectively. Notably, concomitant with suppression of mitochondrial permeabilization, disulfiram dose-dependently inhibited the translocation of GSDMD-NT, GSDME-NT and p-MLKL to mitochondria (**Figure 4D**). Similar results were obtained from J774A.1 macrophages (**Supplementary Figures S2A–D**, **Supplementary Figures S3A–D**). As disulfiram can modify GSDMD-NT to block its oligomerization (20), we assessed whether disulfiram-mediated inhibition of mitochondrial translocation of PANoptotic executors was associated with its inhibition of their oligomerization. As expected, disulfiram reduced GSDMD-NT oligomerization in BMDMs upon induction of PANoptosis, which was revealed by Western blotting under a non-reducing condition (**Figure 4E**). Likewise, the oligomerization of both GSDME-NT and p-MLKL was also decreased by disulfiram treatment (**Figure 4E**). These results together indicated that disulfiram prevented the oligomerization of GSDMD-NT, GSDME-NT and p-MLKL on mitochondria.

**Figure 5:**
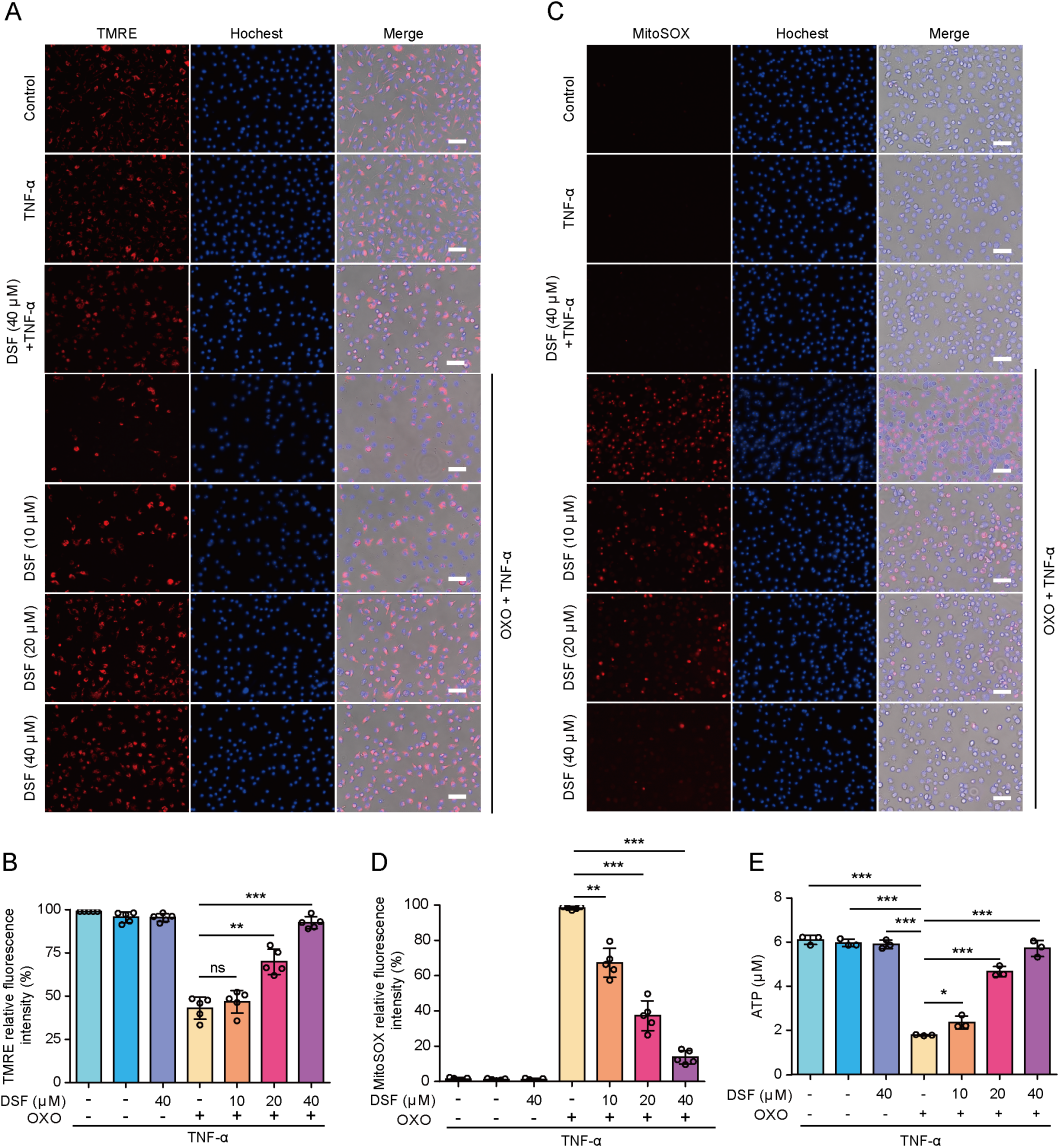
Disulfiram (DSF) prevents the decline of mitochondrial function and ROS production. BMDMs were pretreated with or without DSF as indicated for 0.5 h and then treated with OXO (0.1 μM) for 1 h, followed by stimulation with TNF-α (5 ng/mL) for 2 h in the presence or absence of DSF. **(A, B)** Mitochondrial membrane potential was analyzed by staining with TMRE. Fluorescence images were acquired with fluorescence microscopy **(A)**. Histograms showing the quantitative analysis of TMRE fluorescence **(B)**. **(C, D)** Mitochondrial ROS generation was assayed by staining with MitoSOX **(C)**. Hoechst 33342 was used to reveal nuclei (blue, staining all nuclei). Scale bars, 50 μm. Histograms showing the quantitative analysis of MitoSOX **(D)**. Data are shown as mean ± SD (*n* = 5). **(E)** Histograms showing the intracellular ATP levels. **P < 0.01; ***P < 0.001; ns, not significant.

To further explore whether mitochondrial permeabilization was associated with its functional injury, we assessed the mitochondrial function by staining macrophages with TMRE and MitoSOX, respectively. TMRE staining showed that disulfiram dose-dependently reversed the markedly decreased TMRE fluorescence intensity during PANoptosis induction, reflective of a recovery of mitochondrial function (**Figures 5A, B**). Induction of PANoptosis caused a significant production of mtROS as revealed by MitoSOX fluorescence, while disulfiram attenuated mtROS production in a dose-dependent manner (**Figures 5C, D**). Consistent with mitochondrial injury, intracellular ATP levels were decreased during PANoptosis induction, whereas disulfiram reversed this decrease (**Figure 5E**). Together, these results indicate that inhibition of PANoptosis in macrophages is associated with blockade of mitochondrial permeabilization and dysfunction by preventing PANoptosis executors’ translocation to mitochondria.

### Disulfiram blocks PANoptosome assembly to inhibit PANoptosis signaling

**Figure 6:**
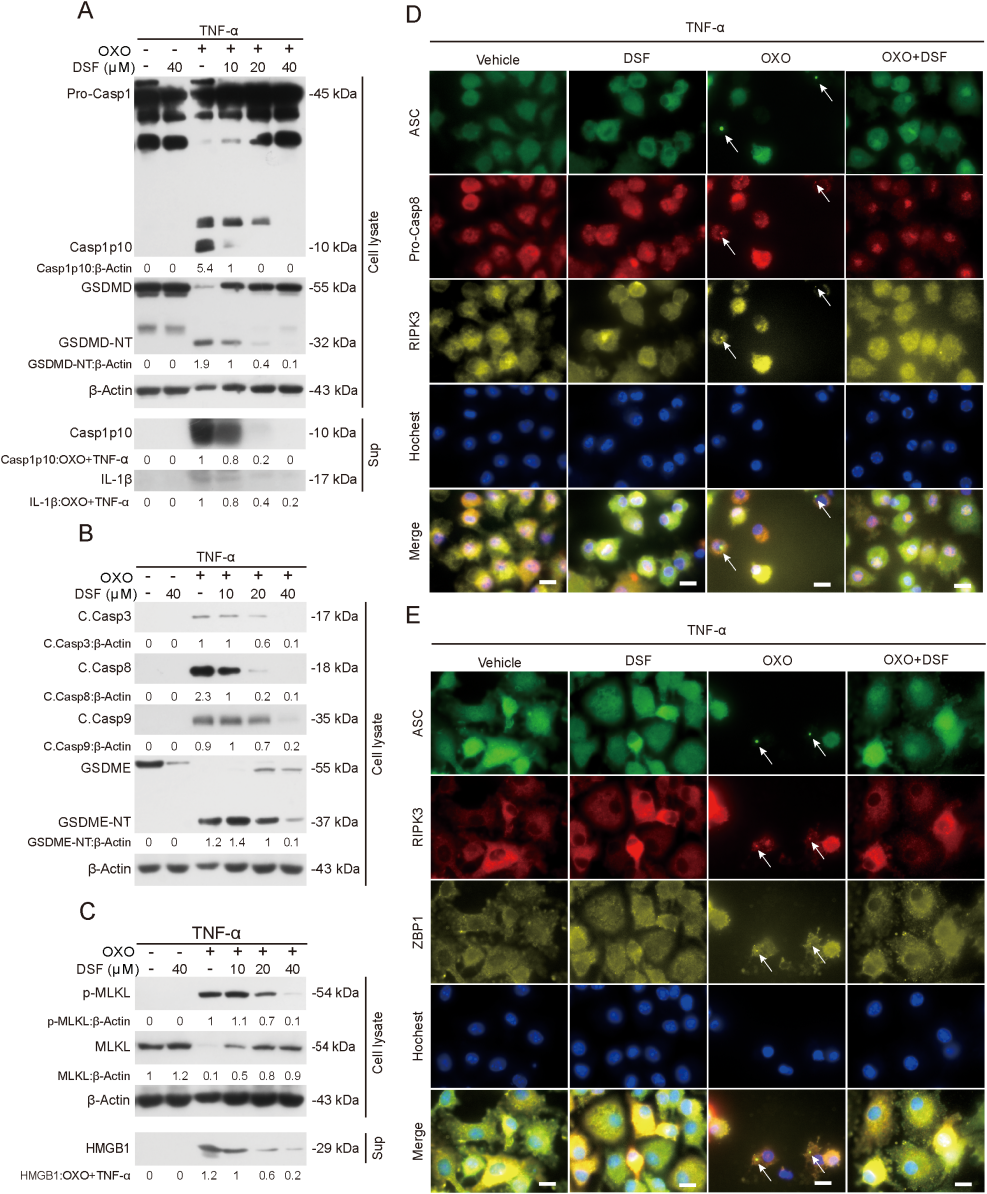
Disulfiram inhibits PANoptotic signaling by blocking the assembly of PANoptosome in macrophages. BMDMs were pretreated with or without disulfiram (DSF) for 0.5 h and then treated with OXO (0.1 μM) for 1 h, followed by stimulation with TNF-α (5 ng/mL) for 2 h in the presence or absence of DSF. **(A–C)** Western blot analysis was used to detect the expression levels of hallmarks for the activation of pyroptosis **(A)**, apoptosis **(B)**, and necroptosis **(C)** signaling in cell lysates or culture supernatants (Sup) of cells. β-Actin was used as a loading control for cell lysates. The values under the blots represent their relative levels. **(D, E)** Immunofluorescence microscopy was adopted to reveal the distribution of ASC, pro-Casp8, and RIPK3 **(D)** or ASC, RIPK3 and ZBP1 **(E)**. Nuclei (blue) were revealed by Hoechst 33342 staining. White arrows indicate ASC specks which were co-localized with puncta of aggregated pro-Casp8, RIPK3, and ZBP1, indicating the assembly of PANoptosome. The images were captured respectively and merged. Scale bars, 10 µm.

Apart from its effect on preventing translocation of GSDMD-NT, GSDME-NT and p-MLKL to mitochondria, we next explored whether disulfiram affected the activation of PANoptotic signaling. To this end, we performed Western blot analysis of cell lysates from macrophages treated with OXO+TNF-α or OXO+LPS. The results showed that the treatment of PANoptosis inducers triggered the activation of pyroptosis (as revealed by generation of caspase-1p10 and GSDMD-NT, as well as the release of caspase-1p10 and mature IL-1β into culture supernatant), apoptosis (as evident by generation of cleaved caspase-3/8/9 and GSDME-NT), and necroptosis signaling (as revealed by phosphorylation of MLKL and release of HMGB1) (**Figures 6A**–**C**). Notably, both these signaling pathways and the release of caspase-1p10, IL-1β and HMGB1 were substantially suppressed by disulfiram treatment (**Figures 6A**–**C**), indicating that disulfiram could inhibit the activation of PANoptotic signaling. Similar results were obtained from BMDMs treated with OXO+LPS (**Supplementary Figures S4A**–**C**) and from J774A.1 cells in response to OXO+TNF-α (**Supplementary Figures S5A**–**C**) or OXO+LPS (**Supplementary Figures S6A**–**C**).

Given previous findings indicating that mitochondrial dysfunction has important roles in initiating and/or promoting PANoptosome assembly (16, 17) and our aforementioned data showing that disulfiram prevented mitochondrial injury during PANoptosis, we next explored whether disulfiram could block the assembly of PANoptosome to inhibit PANoptotic signaling. ASC was distributed relative evenly in control cells, whereas upon induction of PANoptosis by OXO+TNF-α, ASC became aggregated as a speck and ASC speck was colocalized with pro-caspase-8 and RIPK3 aggregates (**Figure 6D**) or with RIPK3 and ZBP1 (**Figure 6E**), indicating the assembly of PANoptosome. Disulfiram was able to block the formation of ASC speck and its colocalization with pro-caspase-8 and RIPK3 aggregates (**Figure 6D**) or with RIPK3 and ZBP1 (**Figure 6E**), indicative of blockade of PANoptosome assembly. The inhibitory effect of disulfiram on PANoptosome assembly was confirmed by Co-IP assay (**Supplementary Figures S7A, B**). As ZBP1 can sense Z-DNA (33) and our previous study showed that Z-DNA has a role in PANoptosome assembly (16), we examined whether Z-DNA was involved in the formation of PANoptosome by using an antibody (Z22) against Z-DNA (34). Immunofluorescence microscopy showed that Z-DNA was induced by OXO+TNF-α treatment as revealed by Z22 staining and Z-DNA appeared to be co-localized with ASC speck and mitochondria, whereas disulfiram was able to attenuate Z-DNA formation and its co-localization with ASC speck (**Supplementary Figure S8A**). Meanwhile, oxidized mtDNA (as revealed by 8-OHdG staining) was detected in cells treated with OXO+TNF-α whereas disulfiram decreased the levels of oxidized mtDNA (**Supplementary Figure S8B**). Disulfiram also dose-dependently diminished the release of mtDNA (**Supplementary Figures S8C, D**). To further assess the role of ZBP1, we knocked down *ZBP1* expression by using siRNA (**Supplementary Figure S9A**). Knockdown of *ZBP1* not only reduced OXO+TNF-α-induced cell death (**Supplementary Figure S9B**) but also reversed OXO+TNF-α-induced mitochondrial injury (**Supplementary Figures S9C**–**F**). In addition, *ZBP1* knockdown blocked the PANoptosome assembly and suppressed Z-DNA formation (**Supplementary Figure S10**), suggesting a role of ZBP1 in sensing and/or stabilizing Z-DNA. Together, these data suggested that disulfiram inhibited PANoptotic signaling by blocking the formation of Z-DNA and the assembly of PANoptosome.

### Disulfiram mitigates inflammation and organ dysfunction in mice of HLH model accompanied by suppression of PANoptosis signaling

**Figure 7:**
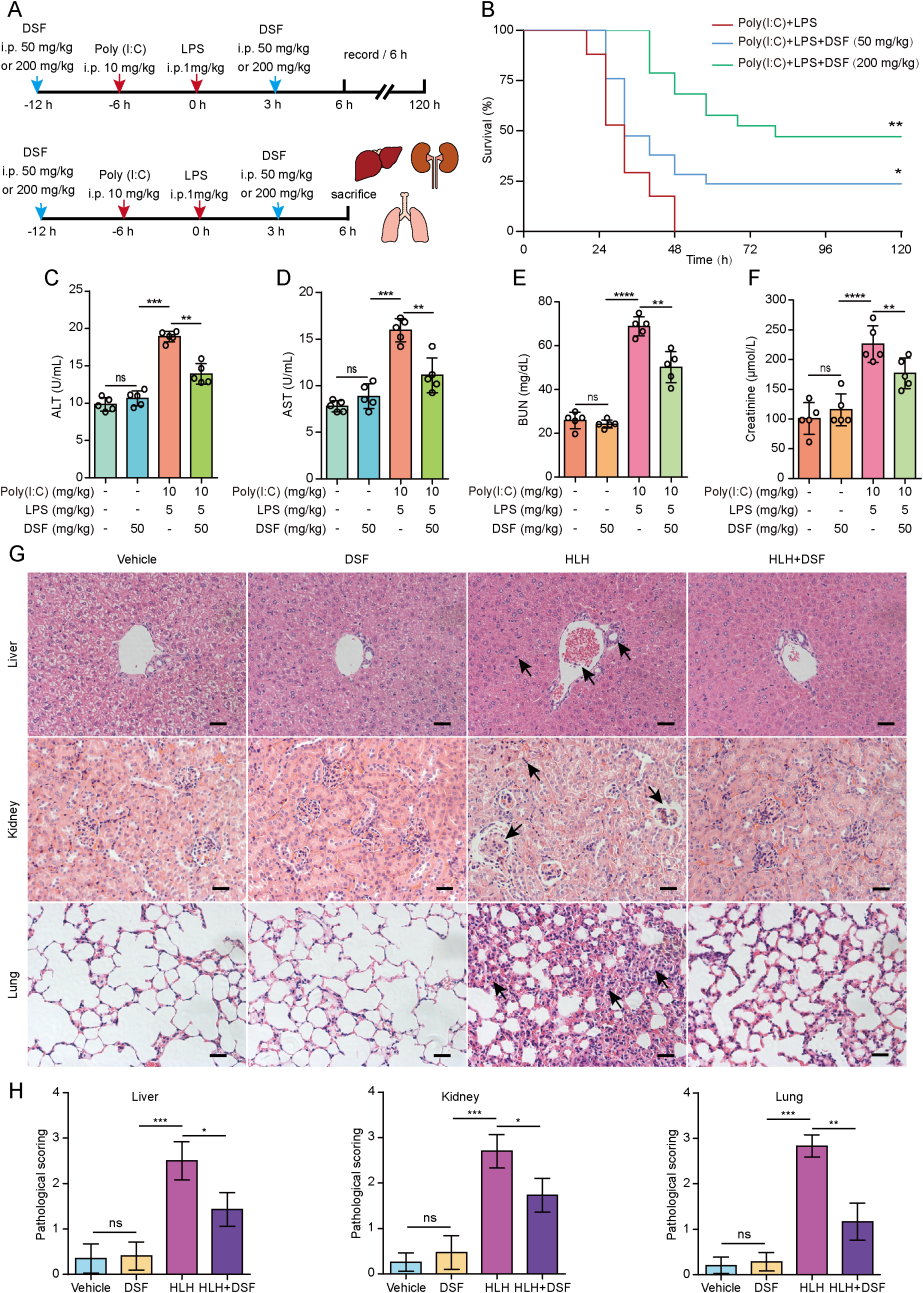
Disulfiram (DSF) increases the survival of mice and mitigates multiple organ injuries in a mouse model of HLH. **(A)** C57BL/6J mice (7–8 weeks of age, 10 mice for each group) were treated with disulfiram (50 mg/kg body weight) or vehicle intraperitoneally (i.p.) and poly(I:C) (10 mg/kg body weight) plus LPS (1 mg/kg body weight) as shown in the schematic for survival (a, upper panel) or for histopathological and biochemical analyses (a, bottom panel). **(B)** Mice survival was monitored every 6 h for 5 consecutive days. Kaplan-Meier survival curves were used to analyze the data (*n* = 10 mice per group). The significance was evaluated by the log-rank (Mantel-Cox) test. *P < 0.05; **P < 0.01 vs poly(I:C)+LPS group. **(C–F)** Serum levels of alanine aminotransferase (ALT) **(c)** and aspartate aminotransferase (AST) **(D)**, blood urea nitrogen (BUN) **(E)**, and creatinine **(F)** in mice treated with disulfiram (50 mg/kg body weight) and poly(I:C)+LPS were measured with respective assay kits. Data are presented as mean ± SD (*n* = 5 mice per group). **P < 0.01; ***P < 0.001; ns, not significant. **(G)** Representative images of the liver, kidney, and lung sections stained with hematoxylin and eosin are shown. Arrows indicate injury areas. Scale bars, 100 μm. **(H)** Histopathological scoring of the liver, kidney and lung. *P < 0.05; **P < 0.01; ***P < 0.001; ns, not significant.

In view of the inhibitory effect of disulfiram on PANoptosis *in vitro*, we sought to assess whether it could mitigate the severity of HLH, which manifests systemic inflammation and multiple organ dysfunction being associated with PANoptosis (9). We established a mouse model of HLH by sequentially intraperitoneal administration of poly(I:C) and LPS as reported previously (9). The hyperinflammation in this mouse model of HLH has be shown to be macrophage intrinsic with natural killer (NK) and T cell being dispensable (35), thus being used to confirm the *in vitro* results from macrophages. Disulfiram was intraperitoneally administered as illustrated in **Figure 7A**. Mice primed with poly(I:C) followed with a challenge of LPS succumbed within two days. However, disulfiram at a dose of 50 mg/kg (body weight) significantly increased the survival of mice to 25% at the end of five days while disulfiram at 200 mg/kg (body weight) increased the survival to 50% (**Figure 7B**). To further delineate organ dysfunction, we evaluated the serum biomarkers for kidney and liver functions in separate experiments 6 h after LPS challenge. The increased ALT and AST serum levels in HLH mice were reduced by disulfiram treatment (**Figures 7C, D**), indicative of attenuated liver injury. The serum BUN and creatinine levels were markedly increased in mice of HLH model, whereas oral administration of disulfiram decreased the levels of these biochemical parameters of HLH mice (**Figures 7E, F**), indicating mitigation of kidney injury of HLH mice. Further, histopathological examination with H&E staining revealed varying degrees of injury in the liver, kidney, and lung of HLH model mice compared to the control group. In the liver of the HLH model group, thrombosis was observed in the portal vein, accompanied by inflammatory cell infiltration and hepatocyte spotty necrosis. The kidneys exhibited significant glomerular atrophy, dilation of Bowman’s capsule, and tubular epithelial cell necrosis. In the lungs, there was prominent perivascular inflammatory cell infiltration, alveolar wall thickening, and alveolar collapse. Notably, disulfiram treatment ameliorated the inflammation and pathological damage in the liver, kidney, and lung of HLH model mice (**Figure 7G**). Subsequently, these tissues were subjected to histopathological scoring by blinded evaluators. The histopathological scores of the liver, kidney, and lung were significantly higher in the HLH model group than in the control group and were significantly lower in the disulfiram treatment group than in the HLH model group (**Figure 7H**).

**Figure 8:**
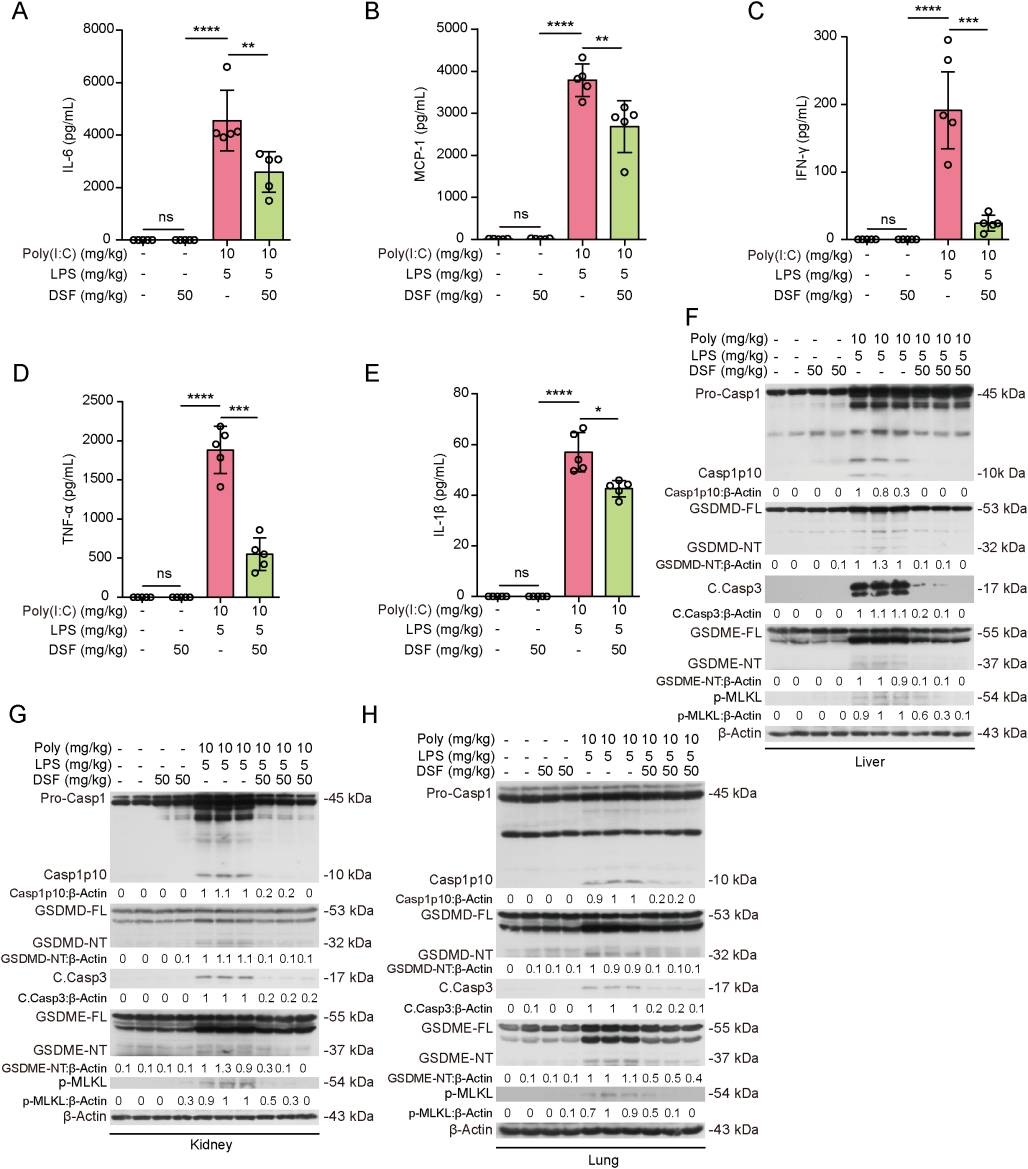
Disulfiram (DSF) mitigates systemic inflammation and PANoptotic signaling in the liver, kidney and lung of mice with HLH. **(A–E)** Mice were treated as shown in **Figure 7A** (bottom panel) and serum IL-6 **(A)**, MCP-1 **(B)**, IFN-γ **(C)**, TNF-α **(D)** and IL-1β **(E)** were quantitatively measured using cytometric bead array (CBA) assays. Data are presented as mean ± SD (*n* = 5 mice per group). *P < 0.05; **P < 0.01; ***P < 0.001; ns, not significant. **(F–H)** Tissue lysates from the liver, kidney and lung were collected respectively. Western blotting analysis was used to examine the expression levels of the activation hallmarks for PANoptosis in the liver **(F)**, kidney **(G)** and lung **(H)** tissue lysates. Samples from 2 mice (for control or DSF alone) or 3 mice (for HLH or HLH+DSF) were analyzed. The values under the blots represent their relative levels. Poly: poly(I:C).

Given the critical role of PANoptosis and inflammation in organ injury of HLH mice (9), we assessed the systemic inflammation and PANoptosis signaling in organs. Measurements of cytokines by cytometric bead array (CBA) showed that several inflammatory cytokines including IL-6, TNF-α, IFN-γ, IL-1β, and MCP-1 (CCL-2) were markedly increased in sera of HLH mice when compared to controls. Disulfiram administration significantly reduced the serum levels of these cytokines (**Figures 8A**–**E**), indicating mitigated systemic inflammation. Previous studies have shown that PANoptosis has a crucial role in the organ injury of HLH and that the combination of TNF-α and IFN-γ has an indispensable role in this process (9). Consistent with these studies, we found that the hallmarks for PANoptotic signaling (caspase-1p10 and GSDMD-NT for pyroptosis, cleaved caspase-3 and GSDME-NT for apoptosis, and p-MLKL for necroptosis) was substantially increased in the liver, kidney and lung of HLH mice while disulfiram treatment reduced the levels of these hallmarks (**Figures 8F**–**H**), suggesting that disulfiram can mitigate systemic inflammation and multiple organ injury by suppressing PANoptosis *in vivo*.

**Figure 9:**
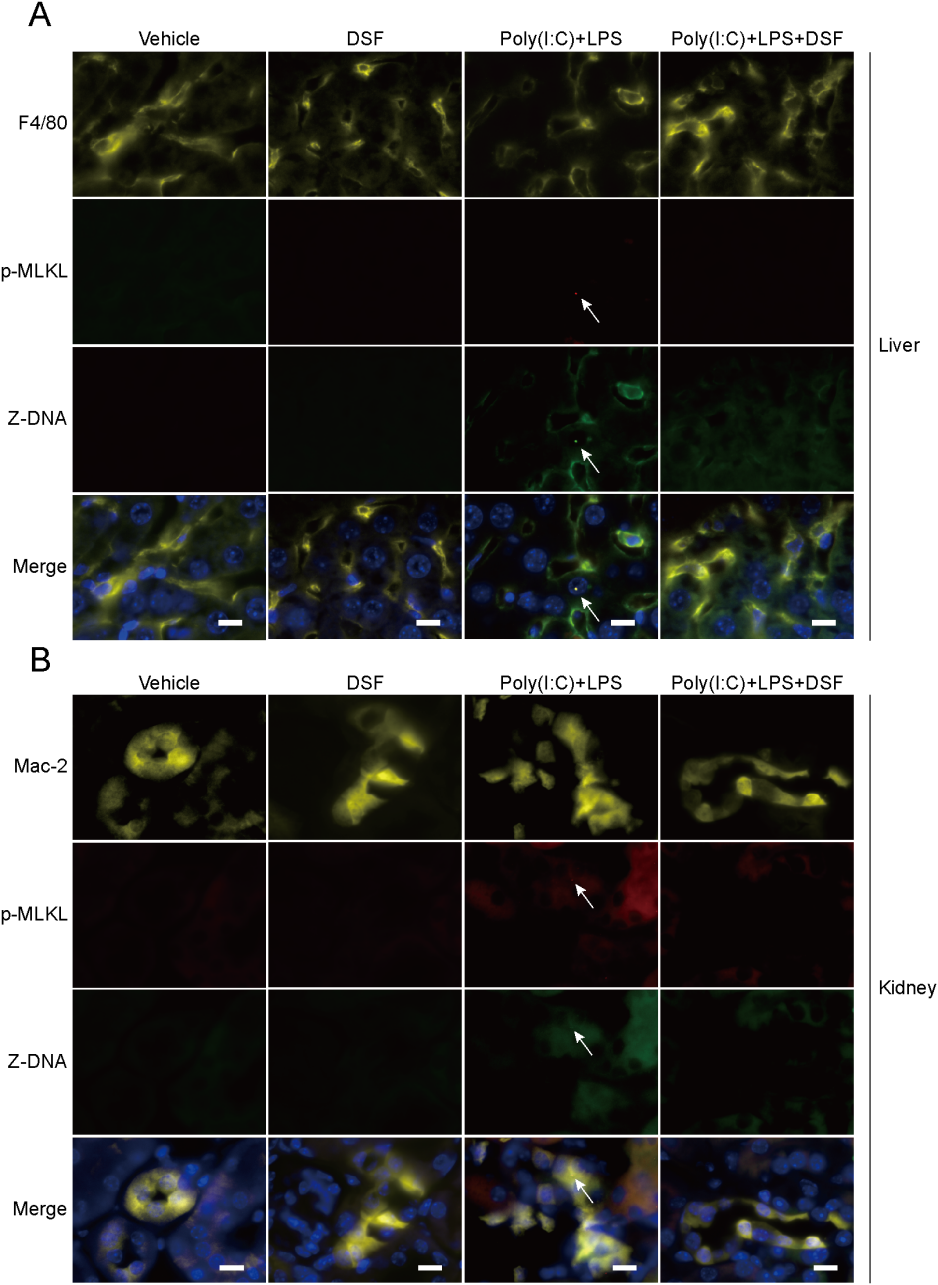
Disulfiram (DSF) inhibits formation of Z-DNA in macrophages of the liver and kidney of mice with HLH. Mice were treated as shown in **Figure 8**. **(A, B)** The liver **(A)** and kidney **(B)** tissues were fixed in 4% paraformaldehyde and frozen sectioned. After heat-induced antigen retrieval, the sections were stained with primary antibodies specific for indicated proteins and appropriate fluorescence secondary antibodies. The tissue sections were covered by antifade mounting medium with DAPI and coverslips. Immunofluorescence images were captured by a fluorescence microscopy. Arrows indicate the co-localization of p-MLKL puncta with Z-NA in liver (F4/80 positive) and kidney (Mac-2-positive) macrophages. Scale bars, 10 µm.

As Z-DNA have a role in PANoptosome assembly (16), we lastly assessed whether some hallmarks of PANoptosome and Z-DNA could be observed *in vivo* in organs of mice with HLH. Indeed, Z22 staining showed that Z-DNA was induced in macrophages (as revealed by F4/80 in the liver and Mac-2 in the kidney and lung) of the liver, kidney and lung of mice treated with poly(I:C) plus LPS and such Z-DNA was co-localized with p-MLKL puncta in macrophages (**Figures 9A, B**, **Supplementary Figure S11**). Disulfiram administration could abrogate Z-DNA formation *in vivo*. These results suggest that Z-DNA may have a role in regulating PANoptotic signaling *in vivo* upon induction of HLH and that disulfiram may suppress PANoptosis signaling by blocking Z-DNA induction.

## Discussion

As a form of lytic cell death, PANoptosis has been shown to play an important role in a wide spectrum of inflammatory diseases including HLH (1, 9). However, the mechanism underlying PANoptotic process remains incompletely understood and drugs targeting this process are an unmet demand (12). In this study, we found that PANoptotic executors GSDMD-NT, GSDME-NT and p-MLKL preferably bound to mitochondria leading to permeabilization of OMM and IMM, which further augmented the lytic cell death during PANoptosis. Importantly, we revealed that the clinically approved anti-alcoholism drug, disulfiram, not only prevented mitochondrial permeabilization concomitant with prevention of PANoptotic executors from binding to mitochondria but also blocked PANoptosome assembly to suppress PANoptotic cell death signaling. Intraperitoneal administration of disulfiram markedly mitigated systemic inflammation and multiple organ injury accompanied by reduced PANoptotic signaling in a mouse model of HLH. Our data may repurpose the anti-alcoholism old-drug disulfiram to treat certain inflammatory diseases that are associated with the activation of PANoptosis signaling.

PANoptosis has been shown to be triggered by a uniquely molecular platform named PANoptosome, which encompasses key components of various regulated cell death pathways including ASC, RIPK3, pro-caspase-8, ZBP1, among others (5–7). A wide range of stimuli has been shown to trigger the assembly of PANoptosome (5), yet the upstream signaling that triggers and/or potentiates this process remains partially understood. Our previous studies revealed that during PANoptosis, oxidized mtDNA could be released and transformed into Z-DNA, the latter of which was further recognized by ZBP1 to trigger PANoptosome assembly (16). The formation of mitochondrial permeability transition pore (mPTP) - and voltage-dependent anion channel (VDAC)-dependent channels in the IMM and OMM, respectively, appeared to be required for mediating mtDNA release and triggering PANoptosome assembly (16). These initial triggers may be crucial in the triggering of PANoptosis signaling; however, it was unclear whether the initially produced PANoptotic executors, including GSDMD-NT, GSDME-NT and p-MLKL, could further induce mitochondrial damage to potentiate PANoptotic cell death.

It has been known that GSDMD-NT binds most strongly to the mitochondrial cardiolipin and to the phosphatidylinositol phosphates (PIPs), PtdIns(4)P and PtdIns(4,5)P2, and less strongly to phosphatidic acid and phosphatidylserine, but does not bind to phosphatidylethanolamine or phosphatidylcholine (28, 29). Similarly, GSDME-NT can strongly bind to cardiolipin (30, 36) and p-MLKL can bind PIPs and cardiolipin as well (31). Of note, cardiolipin is predominantly present on the IMM of mitochondria (37), which is inaccessible from the cytosol. However, small amounts of cardiolipin are reportedly present on OMM under basal conditions and OMM cardiolipin increases during apoptosis (38, 39). Indeed, ~10% of mitochondria isolated from untreated cells have exposed cardiolipin on the OMM, which is accessible to GSDMD-NT (18). It is therefore possible that these small amounts of exposed cardiolipin on OMM provide the initial binding for the PANoptotic executors to form pores and thereby permeabilize mitochondria. Indeed, GSDMD-NT can preferably bind to and permeabilize mitochondria in a cardiolipin-dependent manner (18). GSDME-NT has been reported to permeabilize mitochondria, which appears mediated by cardiolipin (19, 36). In addition, p-MLKL has also been found to translocate to mitochondria to cause mitochondrial injury (40, 41). Consistent with these studies, we found that PANoptotic GSDMD-NT, GSDME-NT and p-MLKL could translocate onto mitochondria preceding lytic cell death, which appeared dependent on cardiolipin because inhibition of cardiolipin synthesis by CRLS1 knockdown diminished mitochondrial translocation. Concurrently, CRLS1 knockdown also reduced mitochondrial damage and lytic cell death during PANoptosis, suggesting an important role of mitochondrial cardiolipin in mediating mitochondrial translocation and permeabilization by these PANoptotic executors.

Mitochondria play critical roles in pyroptosis, apoptosis, and necroptosis (42). In NLRP3 inflammasome-mediated pyroptosis, newly synthesized mtDNA is oxidized by ROS and then fragmented and released through mPTP and VDAC-dependent channels (43–45). The released mtDNA can then trigger the assembly of NLRP3 inflammasome, activation of caspase-1 and generation of GSDMD-NT, leading to pyroptosis (45). During apoptosis, cytochrome *c* is released from the intermembrane space of mitochondria through mitochondrial outer membrane permeabilization (MOMP), leading to apoptosome assembly and eventually apoptotic cell death (42). Mitochondrial ROS has been shown to be involved in necroptosis (46), but the exact underlying mechanism remains to be clarified. Although our previously published data showed that mPTP- and VDAC-dependent channels formed in mitochondria were crucial in initiating PANoptotic signaling (16), we did not know how this signaling was subsequently propagated. In this study, we found that mitochondrial permeabilization by PANoptotic executors had an important role in propagating the initial PANoptotic signaling. The permeabilization of IMM and OMM not only led to the release of ACO2 and cytochrome *c* but also other contents such as PNPT1 and mtDNA. These released mitochondrial components may potentiate PANoptotic signaling by further promoting the assembly of PANoptosome. In support of this notion, our previous study revealed that oxidized mtDNA could be transformed into Z-DNA, which in turn triggered ZBP1-dependent assembly of PANoptosome in macrophages (16). Consistent with this, we did observe Z-DNA formation in our study and such Z-DNA was co-localized with ASC speck. Recognition of Z-DNA by ZBP1 could recruit other proteins, such as RIPK3, to assemble PANoptosome in our experimental setting, which is supported by the results showing that ZBP1 knockdown attenuated PANoptosome formation, mitochondrial injury and cell death. In addition, given the finding that release of PNPT1 (an exoribonuclease in the IMS) has been shown to cause global mRNA decay to promote pyroptosis (18), we presumed that PNPT1 may have a similar function during PANoptosis. Altogether, mitochondrial permeabilization during early phases of PANoptosis has a crucial role in further propagating the cell death signaling.

Considering such a crucial role of mitochondrial permeabilization by GSDMD-NT, GSDME-NT and p-MLKL in PANoptotic cell death, we speculated that prevention of such a process may be a potential way to suppress PANoptotic cell death. As a previous study showing that disulfiram can block GSDMD-NT oligomerization and pore-formation in the plasma membrane (20), we had posited this drug might have similar effects on preventing oligomerization and pore-formation of PANoptotic executors and thus preventing mitochondrial permeabilization. Indeed, our data showed that disulfiram markedly diminished the oligomerization and translocation of GSDMD-NT, GSDME-NT and p-MLKL to mitochondria and prevented the release of both cytochrome *c* (indicative of OMM permeabilization) and ACO2 (indicating IMM permeabilization), suggesting an effective action of disulfiram to protect mitochondria from injury and thereby inhibiting the propagation of PANoptosis.

Cysteine (Cys) residues have been shown to play crucial roles in regulating the activity of GSDMD-NT, GSDME-NT and p-MLKL. Mutations or post-translational modifications of these Cys residues in these proteins can pronouncedly affect their oligomerization and membrane-binding activities, which are important for their pore-forming activity. For instance, mutation of Cys36 or Cys192 to alanine (Ala) in murine GSDMD-NT disrupted its oligomerization (28). Mutation of Cys191 or inhibition of S-palmitoylation of Cys191 in human GSDMD-NT suppresses its membrane localization and dampens pyroptosis (47). A recent study further revealed that simultaneous Cys39/57/192-to-Ala mutations completely abrogate insertion of murine GSDMD-NT insertion into artificial membranes and into the plasma membrane, illustrating a synergistic role of Cys192 palmitoylation-mediated membrane binding and Cys39/57/192-mediated oligomerization in GSDMD-NT pore assembly (48). Moreover, the activity of GSDME could also be regulated by modification of Cys residues via palmitoylation (49) or by succination (50). In addition, phosphorylation of MLKL exposes its 4HB domain to drive the formation of tetramers through intermolecular disulfide bonds (51) and modification of Cys86 of human MLKL by necrosulfonamide inhibits its oligomerization and necroptotic cell death (52). As a Cys-modifying drug that can inactivate a protein by reacting with the sulfhydryl groups of free (reduced) Cys residues (53), disulfiram may be able to modify these Cys residues in PANoptotic executors thereby affecting their activities. Supporting this notion, it has been reported that disulfiram can covalently modify Cys191 (human)/Cys192 (mouse) of GSDMD to inhibit the oligomerization and pore-formation of GSDMD-NT, thereby preventing pyroptosis (20). A recent study further revealed that disulfiram can inhibit NLRP3 palmitoylation at Cys126 residue, thus preventing its localization to the trans-Golgi network and inflammasome activation (21). Consistent with these studies, we found in this study that disulfiram was able to effectively block the oligomerization of GSDMD-NT, GSDME-NT and p-MLKL during PANoptosis, indicating that disulfide bond formation had been abolished. Thus, disulfiram might act as a Cys-modifying drug to react with the Cys residues responsible for disulfide bond formation in these active PANoptotic executors and thereby inhibiting their oligomerization and subsequent mitochondrial translocation and permeabilization. However, we cannot exclude the possibility that disulfiram might also affect cardiolipin distribution on mitochondria or even directly modify mitochondrial proteins. In addition, as disulfiram reacts with the sulfhydryl groups of free (reduced) residues (53), the reaction may be modulated by the cellular redox status. The precise underlying mechanism therefore warrants further investigation.

Apart from blocking PANoptotic executors from permeabilizing mitochondria, we found that disulfiram was also able to inhibit the activation of PANoptotic signaling. This is likely due to that disulfiram-mediated protection of mitochondrial injury had attenuated the release of key components such as cytochrome *c* and mtDNA and thus suppressing the potentiation of cell death signaling by blocking PANoptosome assembly. Supporting this, disulfiram could substantially protect mitochondrial function, block ASC speck formation and its colocalization with pro-caspase-8 and RIPK3 (indicative of abrogation of PANoptosome) and suppress PANoptotic signaling in macrophages during PANoptosis induction. Alternatively, it is also possible that disulfiram could primarily inhibit PANoptosis signaling pathway thereby diminishing mitochondrial damage as a secondary effect, which warrants further clarification.

PANoptosis is a defending mechanism of innate immunity against infections (54), but it also plays important roles in the pathogenesis of inflammatory diseases such as COVID-19 and HLH (1, 9). In these diseases, two key cytokines (i.e., TFN-α and IFN-γ) drives multiple organ injury by induction of PANoptosis, while their neutralization mitigates organ injury in mice treated with TFN-α plus IFN-γ (9). Beyond such pathologies, PANoptosis has been implicated in murine models of inflammatory diseases. For example, poly(I:C) priming followed by LPS challenge in mice can recapitulate most aspects of HLH, providing a robust experimental model (35). In the mouse model of HLH, neutralization of TFN-α and IFN-γ markedly improves survival, thus linking TFN-α+IFN-γ-induced PANoptosis to disease progression (9). Notably, the systemic inflammation in this mouse model mirrors the hyperinflammation observed in murine COVID-19 (9). Our previous studies further demonstrated that all hallmarks for pyroptosis, apoptosis and necroptosis were simultaneously activated in the liver, kidney and lung tissues of poly(I:C)+LPS-induced HLH mice coinciding with multiple organ injury, reconfirming the link between PANoptosis and multiple organ injury in this model (16, 55, 56). Beyond the HLH model, PANoptosis has also been linked to heme (a DAMP) plus a PAMP (such as LPS)-induced pathological organ injury in a mouse model of hemolytic diseases (13). In addition, toxic natural product triptolide induced PANoptosis in murine kidney and liver tissues (11). Considering that the hyperinflammation of poly(I:C)+LPS-induced mouse model of HLH is macrophage-driven with NK and T cells being dispensable (35) and that PANoptosis has been shown to be associated with systemic inflammation and multiple organ damage in the model mice (9, 16, 56), we had chosen this mouse model to verify our *in vitro* disulfiram results derived from macrophages. The dose of disulfiram at 50 mg/kg (body weight) in mice is equivalent to 284 mg/day in humans, which is within the clinically approved range of 125–500 mg/day for treating alcoholism (20, 57). Our data showed that disulfiram administration significantly increased the survival of mice with HLH. Of note, disulfiram mitigated the injury of the liver, kidney and lung, reduced serum levels of cytokines including TNF-α and IFN-γ, and suppressed PANoptotic signaling in these organs, suggesting that disulfiram-mediated inhibition of PANoptosis had a crucial role in attenuating multiple organ injury. Furthermore, we found that macrophages in the liver, kidney and lung of HLH mice exhibited key hallmarks of PANoptosis, suggesting its important roles in causing organ damage. Notably, disulfiram abolished these PANoptotic features in macrophages. This is consistent with the previous study showing that macrophages, but not NK and T cells, drive hyperinflammation in the mouse model of HLH (35).

Although NK cells and T cells have been found to be dispensable in poly(I:C)+LPS-induced mouse model of HLH (35), the actions of disulfiram on other immune cells or on other signaling pathways *in vivo* cannot be ruled out. For example, disulfiram has been reported to suppress antibody-producing reactions by inhibiting macrophage activation and B cell pyrimidine metabolism in a heart transplantation mouse model (58). In addition, disulfiram mitigates STING-dependent inflammation and autoimmunity by targeting RNF115 (59). Interestingly, disulfiram can inhibit the sensing of LPS via TLR4 by modifying MD-2 at Cys133 residue (24). However, the roles of these protein targets of disulfiram in PANoptosis warrant future investigation.

Recently, it has been found that sensing of Z-NA (including Z-RNA and Z-DNA) by ZBP1 has essential roles in pathogenesis of virus infection or other inflammatory conditions. For example, ZBP1-mediated recognition of Z-RNA can induce necroptosis during infection of influenza A virus (IAV) to drive disease severity (60). Sensing of endogenous Z-DNA by ZBP1 has been shown to induce skin inflammation in mice with epidermis-specific RIPK1 knockout and colitis in mice with intestinal epithelial-specific FADD knockout (61). Notably, ZBP1 can stabilize Z-form mtDNA and promote cardiotoxicity of doxorubicin by sensing Z-form mtDNA cooperatively with cyclic GMP-AMP synthase (cGAS) (34). Interestingly, by using the anti-Z-DNA antibody Z22, a recent study showed increased Z-DNA in damaged gut, which could be sensed by ZBP1 to trigger epithelial cell death thereby delaying gut repair (62). Consistent with these reports, we provided initial evidence that Z-DNA could be detected in macrophages of the liver, kidney and lung of mice with HLH accompanying the activation of PANoptosis signaling, suggesting a role for the Z-DNA in PANoptosis and pathogenesis of HLH. Upon recognition of Z-DNA, ZBP1 could trigger the assembly of PANoptosome and subsequently PANoptotic cell death in macrophages and other innate immune cells *in vivo*, leading to the release of cytokines and DAMPs including IL-1β, HMGB1, and ATP (1). These inflammatory cytokines and DAMPs could further act on other immune cells to propagate the inflammatory responses, which ultimately culminated in multiple organ damage in the model mice of HLH. Additionally, ZBP1 activation may also trigger the NF-κB signaling and IRF3 signaling pathways leading to the expression of inflammatory cytokines and type I interferons, respectively (33). Further research is needed to uncover whether these signaling pathways downstream of ZBP1 activation have been involved in pathogenesis of HLH beyond PANoptotic cell death.

In conclusion, we in this study revealed that PANoptotic executors, including GSDMD-NT, GSDME-NT and p-MLKL, bind to and permeabilize mitochondria to potentiate lytic cell death during PANoptosis. The clinically approved anti-alcoholism drug disulfiram can not only prevent the binding of PANoptotic executors to permeabilize mitochondria but also suppress PANoptotic signaling by blocking the assembly of PANoptosome. In a mouse model of HLH, disulfiram administration can mitigate multiple organ injury and systemic inflammation accompanied by diminished PANoptotic signaling. Our data reveal a previously unrecognized activity of disulfiram and repurpose this clinically approved drug for potential treatment of PANoptosis-related inflammatory diseases. However, there are limitations in this study. For instance, the cardiolipin distribution on mitochondria under PANoptosis remains to be explored. Whether cardiolipin knockdown affects apoptosis and necroptosis is also worth investigating. The precise action mechanism of disulfiram on PANoptotic executors is still unclear. It is also unknown whether this Cys-modifying drug can directly interact with mitochondrial membrane through cardiolipin or proteins. The effect of disulfiram on other immune cells during PANoptosis awaits further exploration. Translation of murine disulfiram doses into humans needs future investigation. Resolving these issues will further promote the clinical application of disulfiram.

## Data Availability

The original contributions presented in the study are included in the article/**Supplementary Material**. Further inquiries can be directed to the corresponding authors.
