# Correction: Anti-alcoholism drug disulfiram inhibits PANoptosis by blocking mitochondrial permeabilization in macrophages

**DOI:** 10.3389/fimmu.2026.1782391

**Published:** 2026-01-16

**Authors:** Ya-ping Li, Xin-jian Niu, Ge Zhang, On-kei Chan, Nuo Sun, Bo Hu, Zi-jian Shi, Dong-yun Ouyang, Xian-hui He, Qing-bing Zha

**Affiliations:** 1 Department of Clinical Laboratory, the Sixth Affiliated Hospital of Jinan University (Dongguan Eastern Central Hospital), Dongguan, China; 2 Department of Immunology and Microbiology, College of Life Science and Technology, Jinan University, Guangzhou, China; 3 Department of Nephrology, the First Affiliated Hospital of Jinan University, Guangzhou, China; 4 Department of Fetal Medicine, the First Affiliated Hospital of Jinan University, Guangzhou, China; 5 Center of Reproductive Medicine, the First Affiliated Hospital of Jinan University, Guangzhou, China

**Keywords:** disulfiram, hemophagocytic lymphohistiocytosis, macrophages, mitochondrial permeabilization, PANoptosis

Author Ge Zhang was erroneously omitted as equal contributing author.

The original version of this article has been updated.

