# Supplementary material for: Anti-alcoholism drug disulfiram inhibits PANoptosis by blocking mitochondrial permeabilization in macrophages
Source: Front Immunol. 2025 Dec 19;16:1726408. doi: 10.3389/fimmu.2025.1726408 (PMC12757373; doi:10.3389/fimmu.2025.1726408)
Supplement: Supplementary file 1 [file Table1.docx]

Supplementary materials

**Anti-alcoholism drug disulfiram inhibits PANoptosis by blocking mitochondrial permeabilization and PANoptosome assembly in macrophages**

Ya-ping Li ^1,2, #^, Xin-jian Niu ^2, #^, Ge Zhang ^3^, On-kei Chan ^2^, Nuo Sun ^2^, Bo Hu ^3^, Zi-jian Shi ^4^, Dong-yun Ouyang ^1,2,*^, Xian-hui He ^2,4,*^ and Qing-bing Zha ^1,3,5,*^


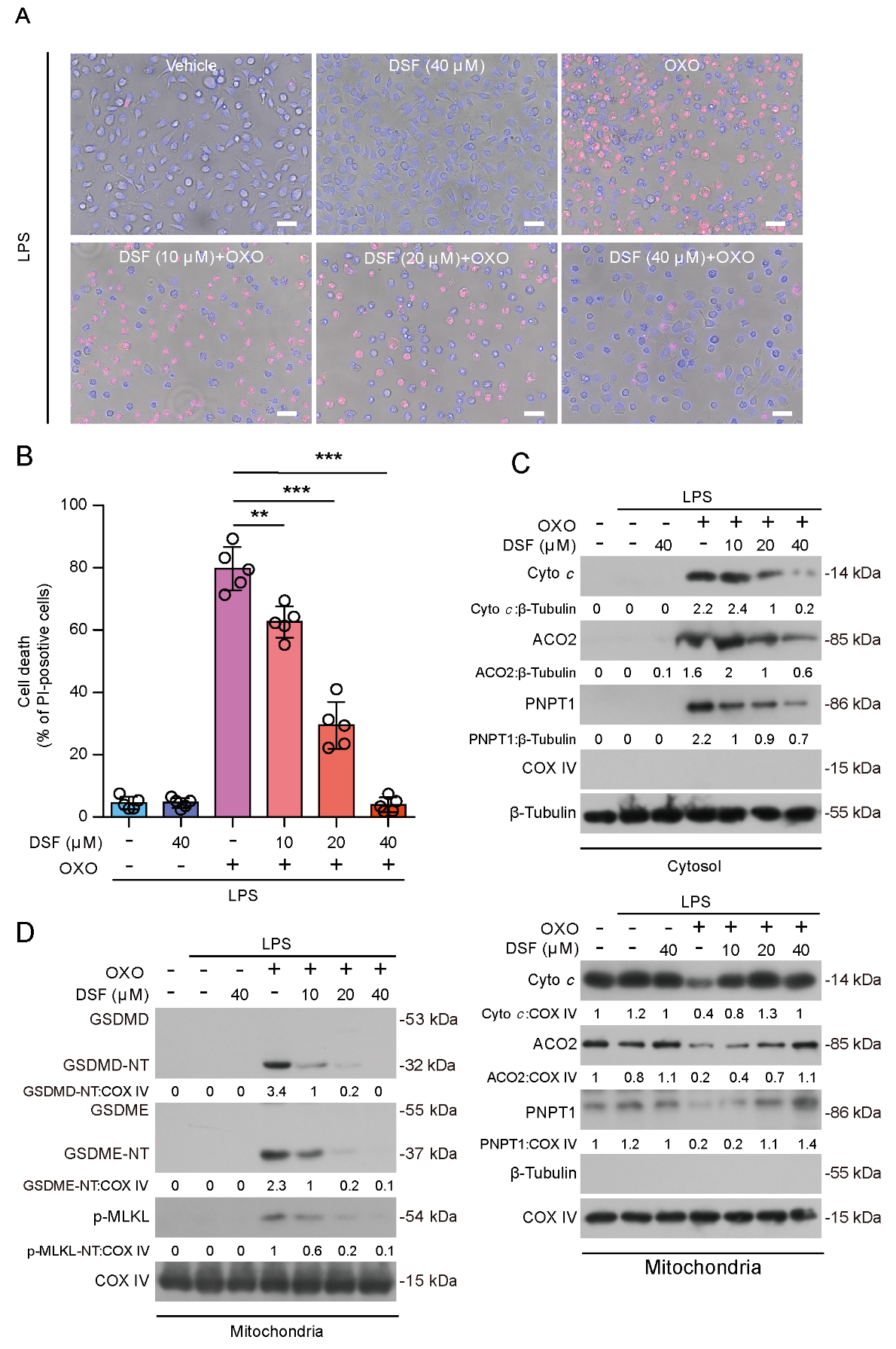


**Figure S1. The inhibition of 5Z-7-oxozeaenol (OXO)+LPS-induced PANoptosis by disulfiram (DSF) is associated with suppression of GSDMD-NT, GSDME-NT, and p-MLKL translocation to mitochondria and mitochondrial permeabilization in macrophages.** Bone marrow-derived macrophages (BMDMs) were pretreated with or without DSF for 0.5 h, and then treated with OXO (0.1 μM) for 1 h, followed by stimulation with LPS (0.5 μg/mL) for 4 h in the presence or absence of DSF. (A, B) Lytic cell death was measured by staining with propidium iodide (PI) (red, staining dying cells) and Hoechst 33342 (blue, staining all nuclei). Fluorescence and bright-field images were captured using fluorescence microscopy (A). Scale bars, 50 μm. Histograms showing quantitative analysis and cell death (B). Data are shown as mean ± SD (*n* = 5). ***P* < 0.01; ****P* < 0.001. (C) The levels of mitochondrial proteins in mitochondria and the cytosol were detected by Western blotting. (D) Western blot analysis of mitochondrial translocation of GSDMD-NT, GSDME-NT, and p-MLKL. β-Tubulin and COV IV were detected as internal controls for the cytosol and mitochondria, respectively. The values under the blots represent their relative levels.


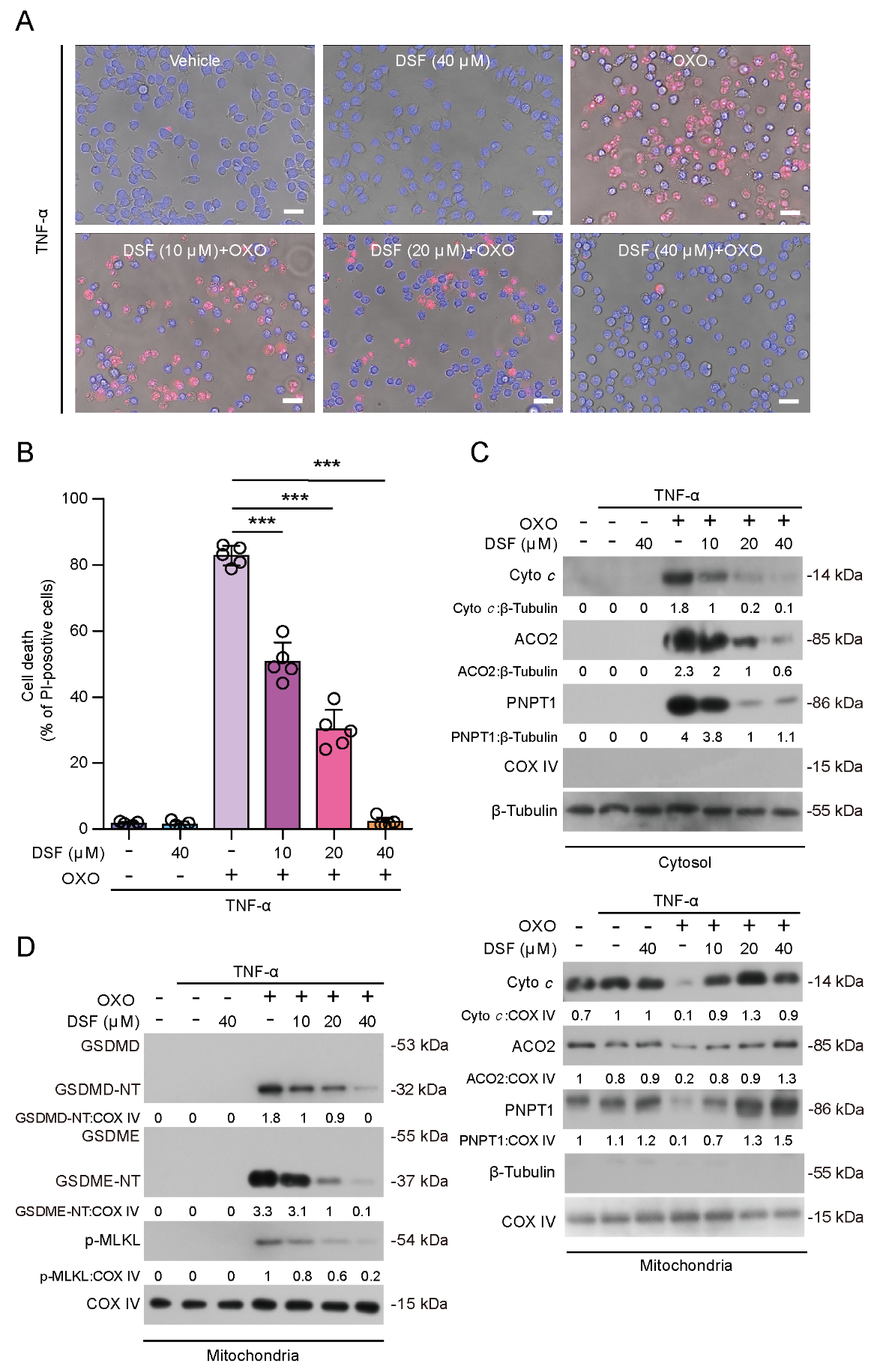


**Figure S2. Inhibition of 5Z-7-oxozeaenol (OXO)+TNF-α-induced PANoptosis by disulfiram (DSF) is associated with suppression of GSDMD-NT, GSDME-NT, and p-MLKL translocation to mitochondria and mitochondrial permeabilization in macrophages.** J774A.1 cells were pretreated with or without DSF for 0.5 h, and then treated with OXO (0.1 μM) for 1 h, followed by stimulation with TNF-α (5 ng/mL) for 3 h in the presence or absence of DSF. (a, b) Lytic cell death was measured by staining with propidium iodide (PI) (red, staining dying cells) and Hoechst 33342 (blue, staining all nuclei). Fluorescence and bright-field images were captured using fluorescence microscopy (A). Scale bars, 50 μm. Histograms showing quantitative analysis and cell death (B). Data are shown as mean ± SD (*n* = 5). ****P* < 0.001. (C) The levels of mitochondrial proteins in mitochondria and the cytosol were detected by Western blotting. (D) Western blot analysis of mitochondrial translocation of GSDMD-NT, GSDME-NT, and p-MLKL. β-Tubulin and COV IV were detected as internal controls for the cytosol and mitochondria, respectively. The values under the blots represent their relative levels.


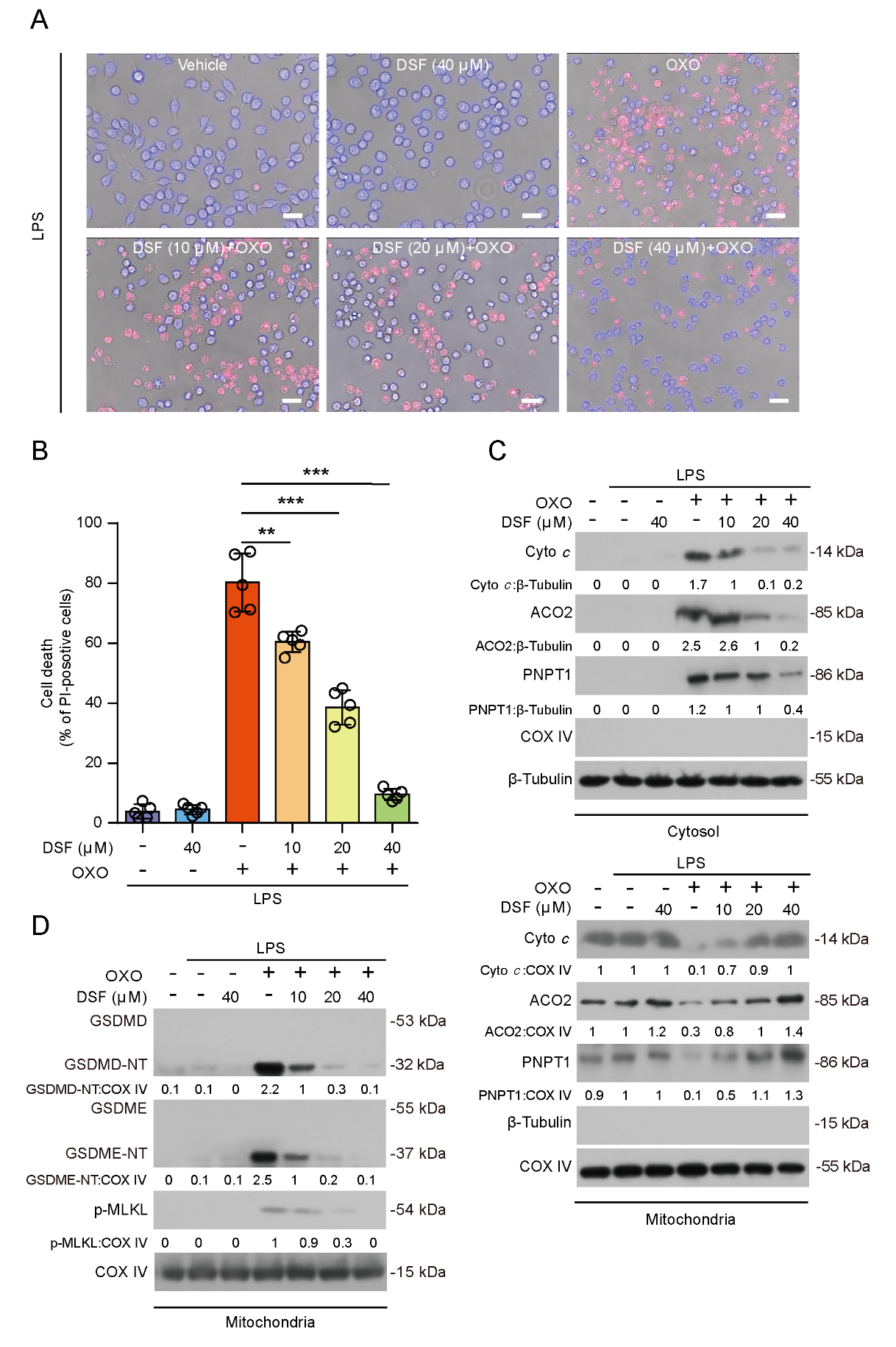


**Figure S3. Inhibition of 5Z-7-oxozeaenol (OXO)+LPS-induced PANoptosis by disulfiram (DSF) is associated with suppression of GSDMD-NT, GSDME-NT, and p-MLKL translocation to mitochondria and mitochondrial permeabilization macrophages.** J774A.1 cells were pretreated with or without DSF for 0.5 h, and then treated with OXO (0.1 μM) for 1 h, followed by stimulation with LPS (0.5 μg/mL) for 5 h in the presence or absence of DSF. (A, B) Lytic cell death was measured by staining with propidium iodide (PI) (red, staining dying cells) and Hoechst 33342 (blue, staining all nuclei). Fluorescence and bright-field images were captured using fluorescence microscopy (A). Scale bars, 50 μm. Histograms showing quantitative analysis and cell death (B). Data are shown as mean ± SD (*n* = 5). ***P* < 0.01; ****P* < 0.001. (C) The levels of mitochondrial proteins in mitochondria and the cytosol were detected by Western blotting. (D) Western blot analysis of mitochondrial translocation of GSDMD-NT, GSDME-NT, and p-MLKL. β-Tubulin and COV IV were detected as internal controls for the cytosol and mitochondria, respectively. The values under the blots represent their relative levels.


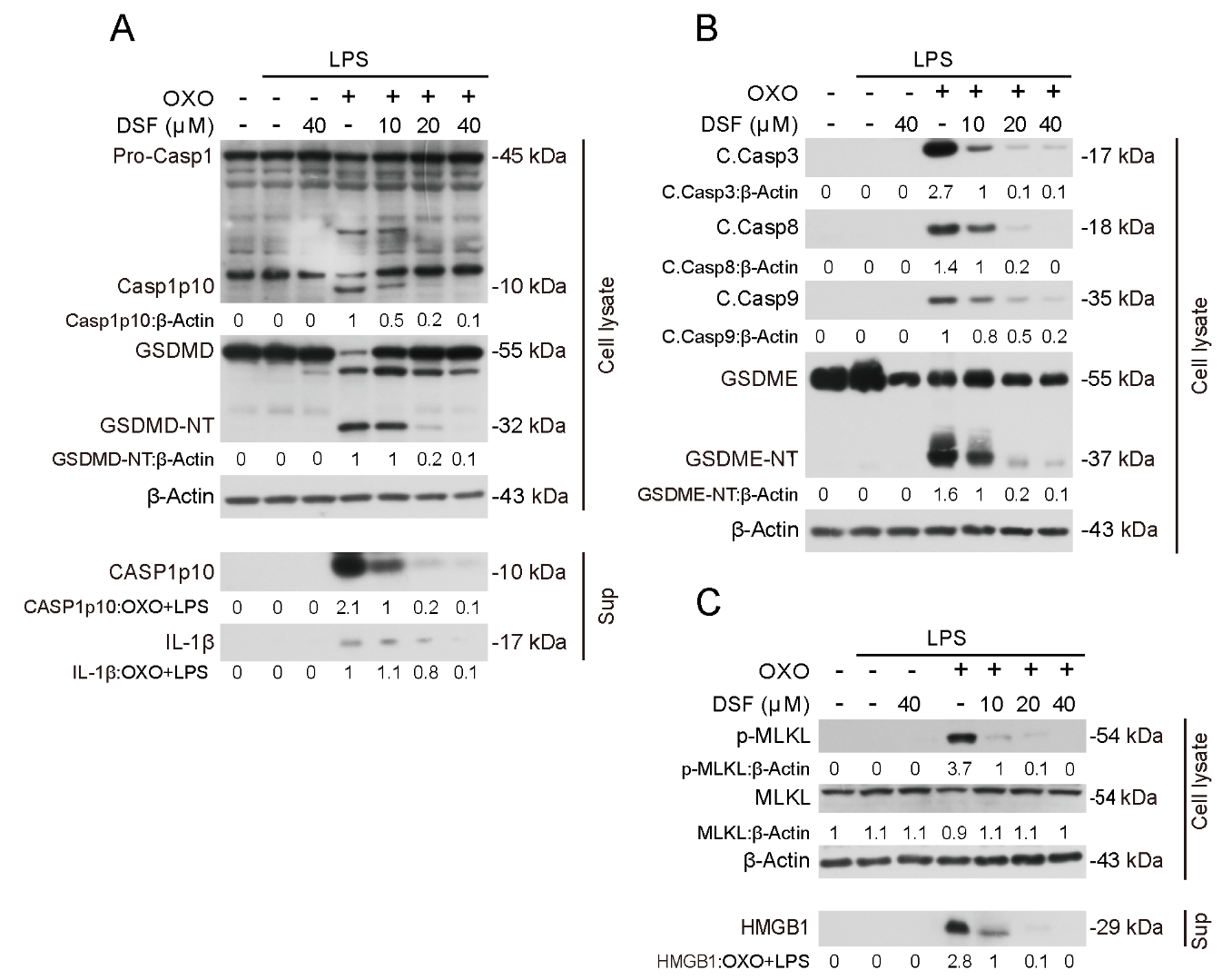


**Figure S4.** **The inhibition of OXO+LPS-induced PANoptosis by disulfiram (DSF) is associated with suppression of GSDMD-NT, GSDME-NT, and p-MLKL generation in macrophages.** BMDMs were pretreated with or without DSF for 0.5 h, and then treated with OXO (0.1 μM) for 1 h, followed by stimulation with LPS (0.5 μg/mL) for 4 h in the presence or absence of DSF. (A-C) Western blot analysis was used to detect the expression levels of hallmarks for the activation of pyroptosis (A), apoptosis (B), and necroptosis (C) signaling in cell lysates or culture supernatants (Sup) of BMDMs. β-Actin was used as a loading control for cell lysates. The values under the blots represent their relative levels.


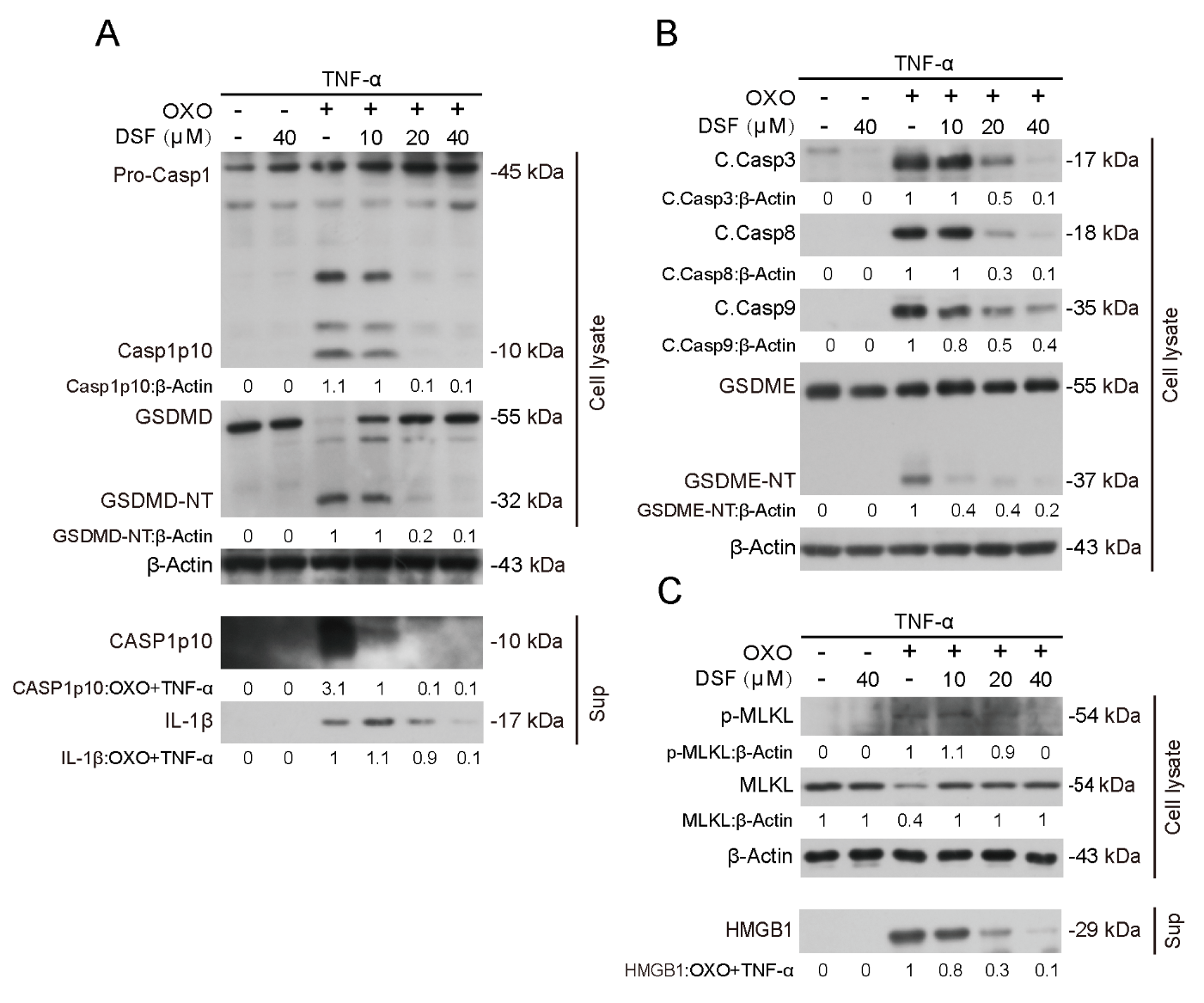


**Figure S5.** **Inhibition of OXO+TNF-α-induced PANoptosis by disulfiram (DSF) is associated with suppression of GSDMD-NT, GSDME-NT, and p-MLKL generation in macrophages.** J774A.1 cells were pretreated with or without DSF as indicated for 0.5 h, and then treated with OXO (0.1 μM) for 1 h, followed by stimulation with TNF-α (5 ng/mL) for 3 h in the presence or absence of DSF. (A-C) Western blot analysis was used to detect the expression levels of hallmarks for the activation of pyroptosis (A), apoptosis (B), and necroptosis (C) signaling in cell lysates or culture supernatants (Sup) of BMDMs. β-Actin was used as a loading control for cell lysates. The values under the blots represent their relative levels.


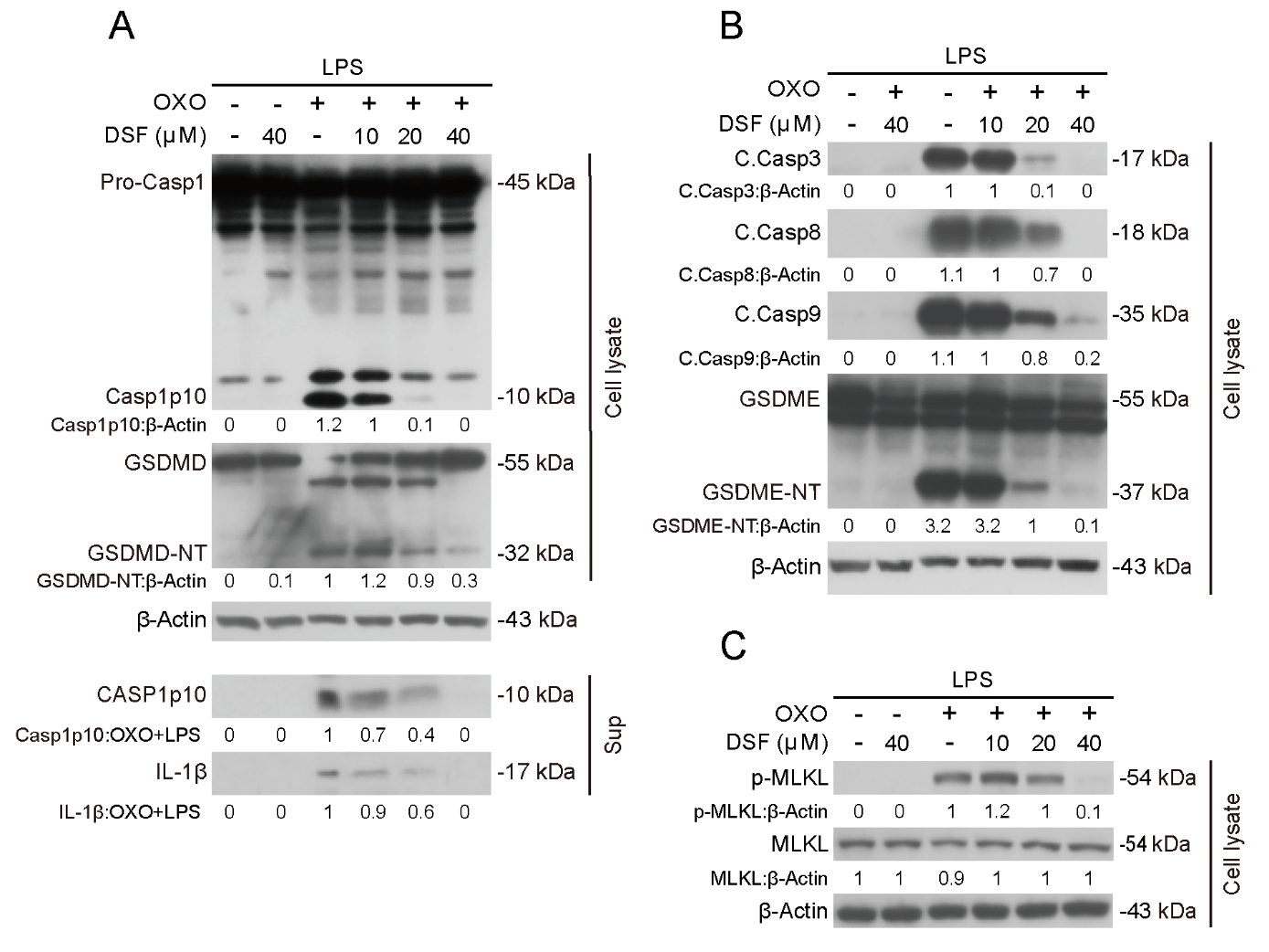


**Figure S6. The inhibition of OXO+LPS-induced PANoptosis by disulfiram (DSF) is associated with suppression of GSDMD-NT, GSDME-NT, and p-MLKL generation in macrophages.** J774A.1 cells were pretreated with or without DSF as indicated for 0.5 h, and then treated with OXO (0.1 μM) for 1 h, followed by stimulation with LPS (0.5 μg/mL) for 5 h in the presence or absence of DSF. (A-C) Western blot analysis was used to detect the expression levels of hallmarks for the activation of pyroptosis (A), apoptosis (B), and necroptosis (C) signaling in cell lysates or culture supernatants (Sup) of BMDMs. β-Actin was used as a loading control for cell lysates. The values under the blots represent their relative levels.


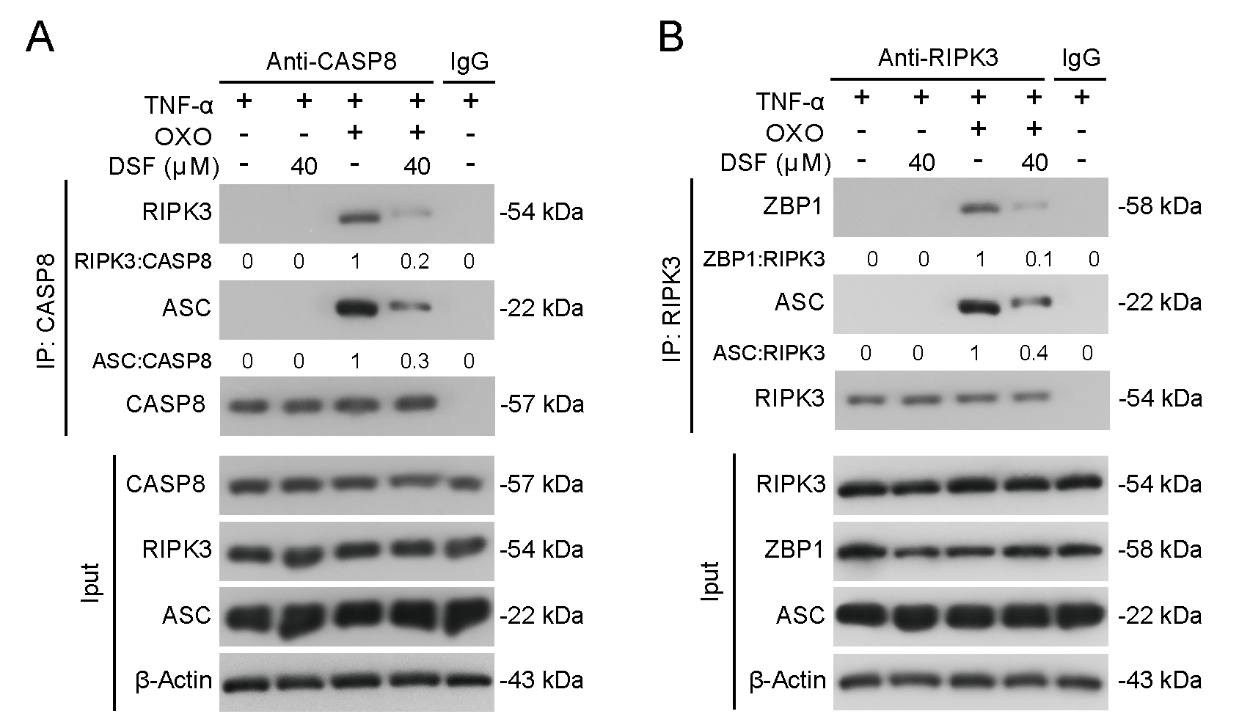


**Figure S7. Disulfiram inhibits the interactions between key components of PANoptosome during PANoptosis.** BMDMs were pre-treated with or without disulfiram (DSF) for 0.5 h, followed by treatment with OXO (0.1 μM) and TNF-α (5 ng/mL) for 2 h in the presence or absence of DSF. The interactions between CASP8 and RIPK3/ASC, as well as between RIPK3 and ASC/ZBP1 following the indicated treatments were evaluated by co-immunoprecipitation assay. The indicated protein levels were detected by Western blotting. The values under the blots represent their relative levels.


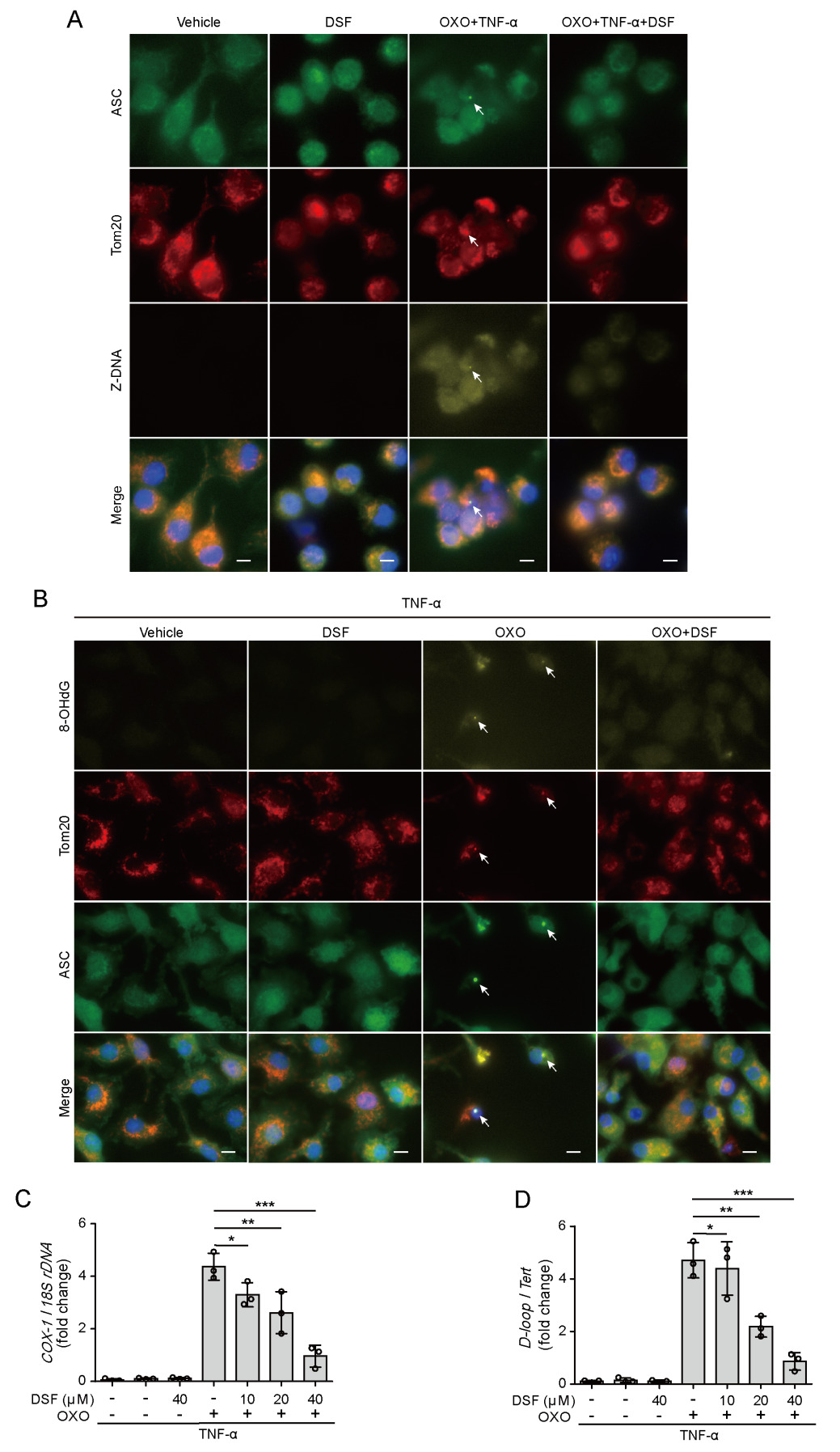


**Figure S8. OXO+LPS-induced Z-DNA during PANoptosis is co-localized with PANoptosome and is abrogated by disulfiram (DSF).** BMDMs were pretreated with or without DSF for 0.5 h, and then treated with OXO (0.1 μM) for 1 h, followed by stimulation with TNF-α (5 ng/mL) for 2 h in the presence or absence of DSF. (A, B) Immunofluorescence microscopy was used to reveal the distribution of ASC, Tom20, and Z-DNA (A) or 8-OHdG, ASC, and Z-DNA (B). Nuclei (blue) were revealed by Hoechst 33342 staining. Arrows indicate ASC specks which were co-localized with puncta of Tom20 or 8-OHdG and Z-DNA, indicating the assembly of PANoptosome. The images were captured respectively and merged. Scale bars, 10 µm. (C, D) Mitochondrial DNA levels in the cytosol were determined by quantitative PCR (qPCR). **P* < 0.05; ***P* < 0.01; ****P* < 0.001.


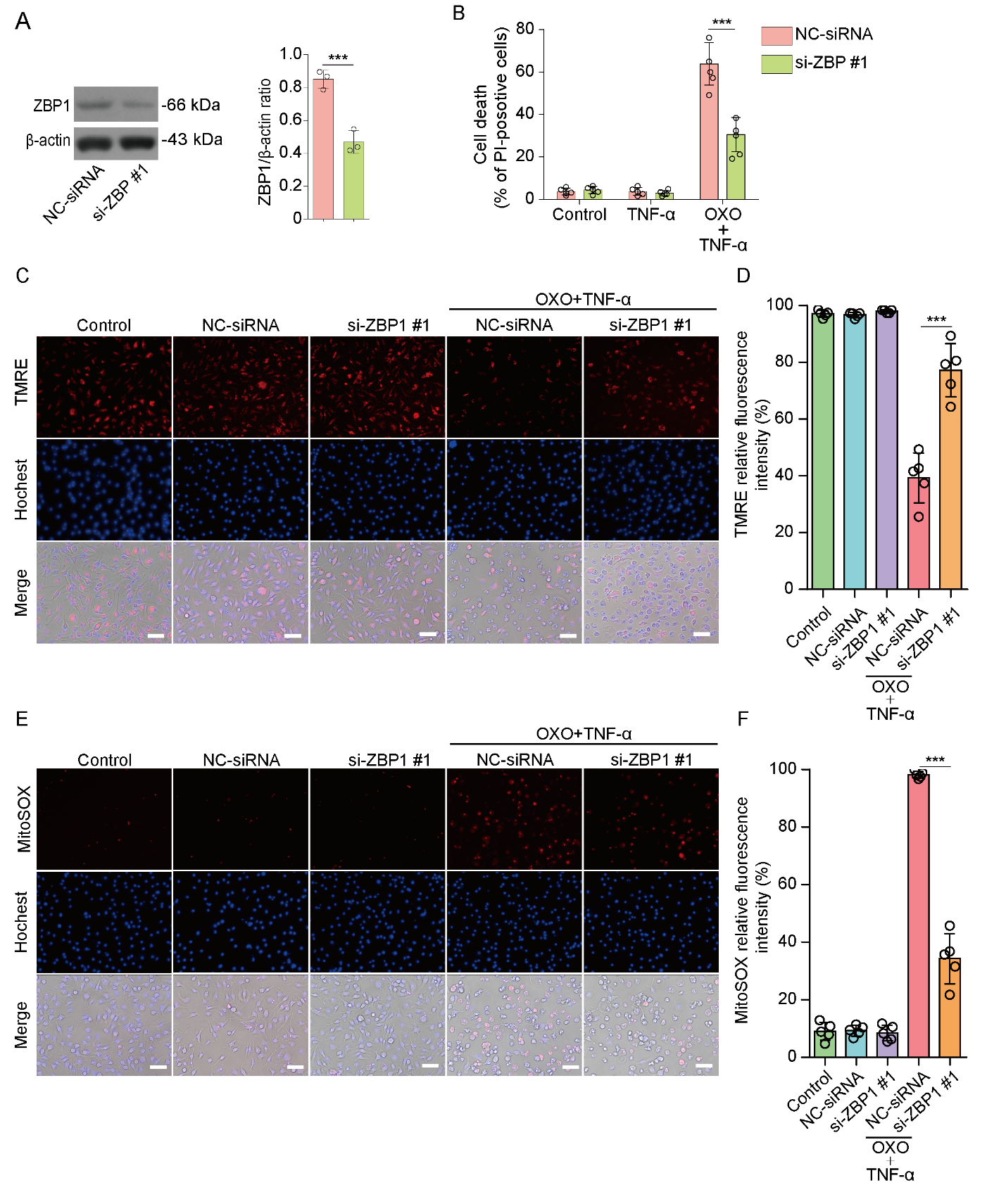


**Figure S9. Knockdown of *ZBP1* attenuates PANoptotic cell death and mitochondrial dysfunction in macrophages.** *ZBP1* knockdown in BMDMs was performed using specific siRNA (si-ZBP1 #1) with NC-siRNA as control. Western blot analysis of ZBP1 expression was conducted 48 h post-transfection (A, left panel). Knockdown efficiency was quantified after normalization to β-actin, with data presented as mean ± SD (a, right panel). (B) PI-positive cells in 5 randomly chosen fields were quantified and percentage of cell death is defined as the ratio of PI-positive over all cells (revealed by Hoechst 33342). Mitochondrial membrane potential was evaluated by TMRE staining (C, D). Mitochondrial ROS production was measured using MitoSOX staining (E, F). All images were acquired by fluorescence microscopy, with quantitative data shown as histograms (mean ± SD, *n* = 5). Scale bars, 50 μm. ****P* < 0.001.


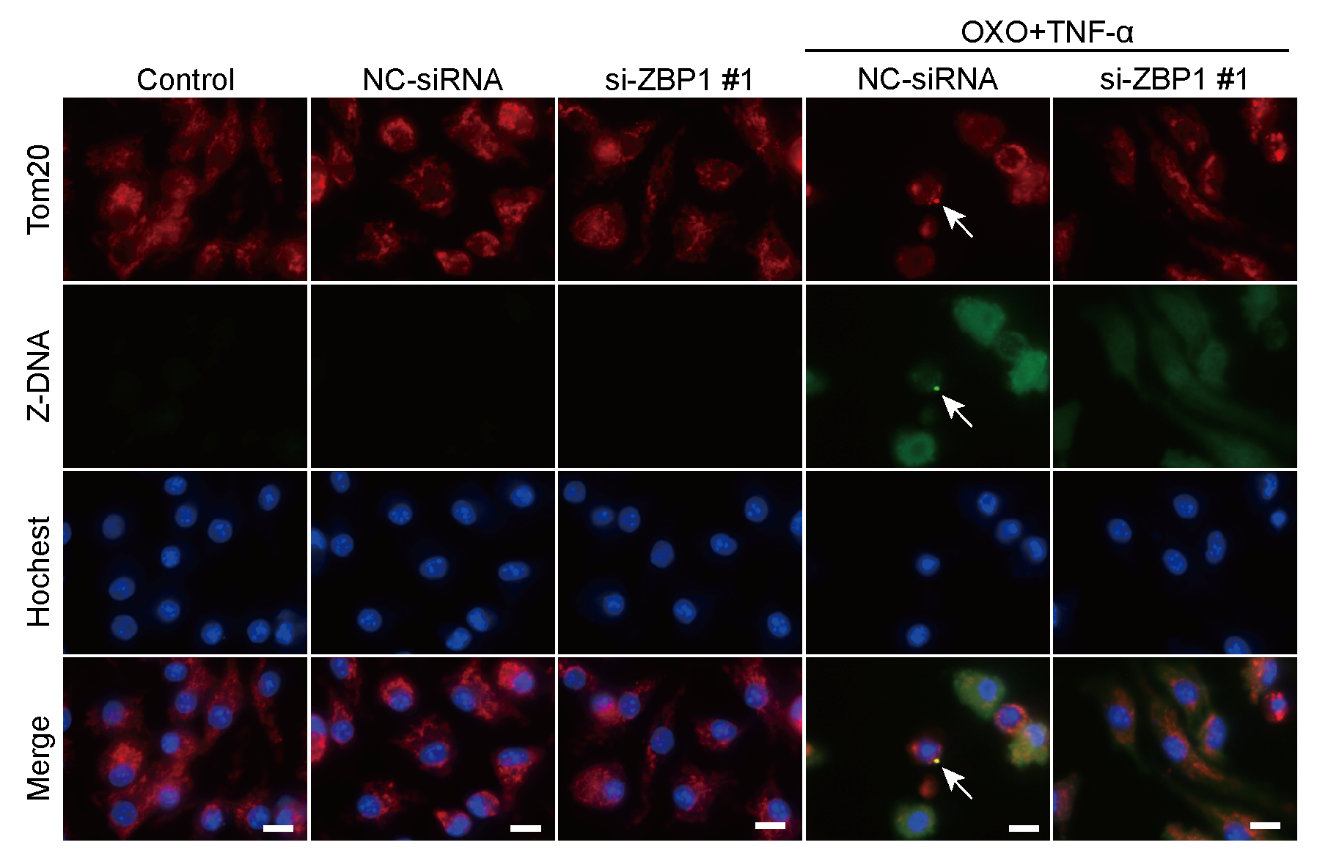


**Figure S10. Knockdown of *ZBP1* attenuates Z-DNA formation and PANoptosome assembly during PANoptosis.** Following *ZBP1* knockdown in BMDMs using specific siRNA (si-ZBP1 #1), cells were treated with OXO (0.1 μM) for 1 h followed by TNF-α (5 ng/mL) stimulation for 2 h. The subcellular distribution of Tom20 (red) and Z-DNA (green) was examined by immunofluorescence microscopy. Nuclei were counterstained with Hoechst 33342 (blue). White arrows indicate co-localization of Z-DNA punctum with Tom20 aggregates, suggesting PANoptosome assembly. Individual channel images were acquired separately and subsequently merged. Scale bars, 10 μm.


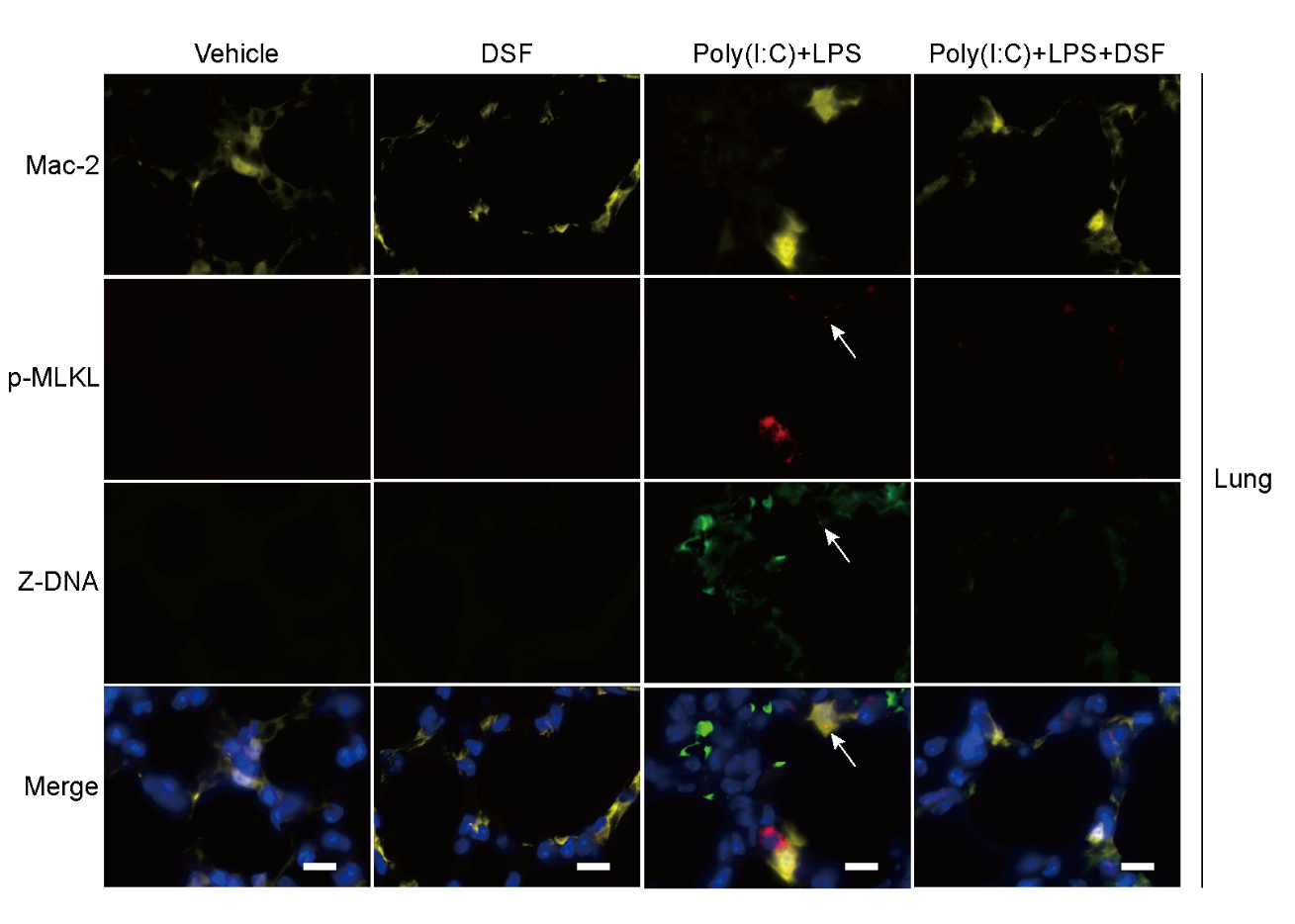


**Figure S11. Disulfiram (DSF) inhibits formation of Z-DNA in macrophages of the liver and kidney of mice with HLH.** Mice were treated as shown in Figure 8. The lung tissues were fixed in 4% paraformaldehyde and frozen sectioned. After heat-induced antigen retrieval, the sections were stained with primary antibodies specific for indicated proteins and appropriate fluorescence secondary antibodies. The tissue sections were covered by antifade mounting medium with DAPI and coverslips. Immunofluorescence images were captured by a fluorescence microscopy. Arrows indicate the co-localization of p-MLKL puncta with Z-NA in macrophages (Mac-2 positive). Scale bars, 10 µm.
